# RNAseq of Deformed Wing Virus and Other Honey Bee-Associated Viruses in Eight Insect Taxa with or without *Varroa* Infestation

**DOI:** 10.3390/v12111229

**Published:** 2020-10-29

**Authors:** Laura E. Brettell, Declan C. Schroeder, Stephen J. Martin

**Affiliations:** 1 Hawkesbury Institute for the Environment, Western Sydney University, Locked bag 1797, Penrith, NSW 2751, Australia; 2 School of Environment and life Sciences, University of Salford, Manchester M5 5WT, UK; s.j.martin@salford.ac.uk; 3 Veterinary Population Medicine, College of Veterinary Medicine, University of Minnesota, St Paul, MN 55108, USA; dcschroe@umn.edu; 4 School of Biological Sciences, University of Reading, Reading RG6 6LA, UK

**Keywords:** Deformed wing virus, virome, hymenoptera, honey bee, *Varroa*, spillover, viruses

## Abstract

The global spread of a parasitic mite (*Varroa destructor)* has resulted in Deformed wing virus (DWV), a previously rare pathogen, now dominating the viromes in honey bees and contributing to large-scale honey bee colony losses. DWV can be found in diverse insect taxa and has been implicated in spilling over from honey bees into associated (“apiary”) and other (“non-apiary”) insects. Here we generated next generation sequence data from 127 insect samples belonging to diverse taxa collected from Hawaiian islands with and without *Varroa* to identify whether the mite has indirectly affected the viral landscapes of key insect taxa across bees, wasps, flies and ants. Our data showed that, while *Varroa* was associated with a dramatic increase in abundance of (predominantly recombinant) DWV in honey bees (and no other honey bee-associated RNA virus), this change was not seen in any other taxa sampled. Honey bees share their environment with other insect populations and exist as a homogenous group, frequently sharing common viruses, albeit at low levels. Our data suggest that the threat of *Varroa* to increase viral load in an apiary does not automatically translate to an increase in virus load in other insects living in the wider community.

## 1. Introduction

In the 1950s, the ectoparasitic *Varroa* mite (*Varroa destructor*), jumped from its native host the Asian honey bee, *Apis cerana* to the European honey bee, *Apis mellifera* [1], the most commonly managed bee species around the world. The widespread geographic range and extensive global commercial movement of this species meant that the mite was able to quickly establish in honey bee populations across the globe [2], where it has been responsible for large scale colony losses, due in large part to its efficiency as a vector of honey bee-associated viruses, most notably deformed wing virus (DWV) [3]. Nowhere has this catastrophic association between *Varroa*, DWV and colony loss been more evident than in Hawaii [4]. Our previous work [4] identified that not only did the prevalence and titre of DWV increase in the honey bee population as *Varroa* became established, but also the genetic variation of DWV dramatically decreased, a phenomenon independently confirmed by Ryabov et al. [5] in the UK. In Hawaii, the islands which experienced *Varroa* infestations and high DWV loads correspondingly experienced substantial honey bee colony losses, in both managed bees as well as feral populations. It has become clear that DWV naturally exists at very low levels as a highly diverse cloud of variants. The change in transmission—i.e., the feeding of *Varroa* mites, has selected for a small group of these genetic variants to proliferate of which three master variants (DWV-A, -B (previously Varroa destructor virus-1, VDV-1), and -C) have been described [6,7,8] and two of which (DWV-A and DWV-B) are now prevalent in honey bee colonies worldwide [3,9].

Following concerns about insect declines, widespread sampling has detected DWV in a diverse range of hosts, including over 65 arthropod species spanning eight insect orders and three Arachnida orders (reviewed in [10]). The presence of DWV in these insects is largely associated with its prevalence and load in local honey bee populations [11,12,13,14], suggesting that honey bees are the reservoir of DWV that is threatening the wider arthropod community via spillover of the virus [15,16]. Other studies have also used the Hawaiian system and have showed that, on islands where *Varroa* was present, DWV prevalence in hymenopteran species of paper wasps (*polistes* spp.) and a solitary bee *Ceratina smaragdula* increases compared to islands without *Varroa*, mirroring the situation in the honey bees [14]. Additionally, Brettell et al. [17] showed that DWV was frequently detected in pest species that share the same space as honey bees, such as social wasps, hive beetles and ants, but variations in genetic variants between the bees and pest species existed, indicating that the situation was more complex than a simple spillover event. Loope at al. [18], did not see any change in DWV prevalence or abundance in the yellowjacket wasp (*Vespula pensylvanica*), before and after *Varroa* became established on Big Island (Hawaii). However, they did find that DWV viral diversity was significantly reduced following *Varroa* introduction, mirroring what Martin et al. [4] and Ryabov et al. [5] found in honey bees. This suggests that DWV may be a generalist insect virus, but now honey bee-selected variants driven by *Varroa* transmission are spilling back into a more diverse range of insects, via a range of interactions with honey bees, such as predation, robbing and sharing foraging sites. Although studies have confirmed viral replication in bumblebees through infection experiments [19,20], the majority of studies to date, which have investigated viral spillover from honey bees to other taxa [12,14,21,22], have used an RT-PCR-based approach and although this has revealed valuable information, there may be unrealized virus diversity missed with such primer-dependent methods. In addition, the critical viral load data are often lacking, so it is difficult to assess the impact of the virus simply by its presence.

The aim of this study was to generate Next Generation Sequence data (RNAseq) to identify if the presence of *Varroa* in the honey bee population is associated with shifts in the RNA viral landscape of diverse insect hosts; namely, honey bees (Apis), solitary bees, a yellowjacket wasp (*Vespula pensylvanica*), potter wasps (Eumeninae), paper wasps (*Polistes)*, solitary wasps, ants (Formica) and flies (Diptera). In addition, the diversity of honey bee-associated viruses in each species, as well as an indication of viral load, relative to honey bees, was investigated.

## 2. Materials and Methods 

### 2.1. Experimental Setup

To determine whether the presence of *Varroa* in honey bee populations is impacting the wider insect community, we collected representative samples of diverse insect taxa from islands with established *Varroa* populations (>5 yrs) (Oahu and Big Island) and islands where honey bee populations have never been exposed to *Varroa* (Kauai and Molokai). Within these sites, to indicate whether there was an effect of proximity to managed honey bees, sample sites were designated “apiary”—comprising sites either on, or in close proximity to known apiary locations (<2 km)—or “non-apiary”—samples sites away from known apiary sites (>10 km). To capture a wide diversity of taxa common across the Hawaiian archipelago, insects were collected and assigned to one of eight taxon groups; honey bees (Apis), solitary bees, a yellowjacket wasp (*Vespula pensylvanica*), potter wasps (Eumeninae), paper wasps (*Polistes)*, solitary wasps, ants (Formica) and flies (Diptera). Taxonomic identifications were made morphologically and are given for each sample in Table S1. Four samples were chosen at random per treatment (with *Varroa*/apiary, with *Varroa*/non-apiary, no *Varroa*/apiary, no *Varroa*/non-apiary) for each group. Samples were collected as individuals, with the exception of honey bees which were in pools of five (to obtain a more general picture of the honey bee viral landscape) and ants which were also in pools of five, due to their small size. All insects were collected while foraging, with the exception of apiary honey bee samples which were collected from hive entrances. Samples were collected using a net or pooter into sterile tubes and transferred back to the laboratory on ice, where they were identified morphologically before storing in 70% ethanol at 4 °C. Collections were made during November 2015, March–May 2016 and October 2016.

### 2.2. Sample Preparation and RNA Extraction

Prior to total RNA extraction, samples were briefly dried to remove excess alcohol followed by homogenization using a sterile pestle and mortar, or minipestle and microcentrifuge tube, and Liquid nitrogen. Depending on the size of the insect, either whole individuals, pools, or an aliquot of homogenate was used for RNA extraction (Table S1), which was carried out using the RNeasy mini kit following manufacturer’s recommendations, eluting in 30 µL nuclease-free water. RNA was then quantified by spectrophotometry (Nanodrop, Thermo Fisher Scientific, Loughborough, UK) and stored at −80 °C.

### 2.3. cDNA Library Preparation

RNA samples were DNase treated using the RQ1 RNase free DNase kit (Promega, Southampton, UK) following the manufacturer’s instructions and re-quantified (Nanodrop, Thermo Fisher Scientific). cDNA libraries were created using the NEBNext Ultra RNA Library prep kit with the NEBNext Poly(A) mRNA isolation module and NEBNext Multiplex Oligos (New England Biolabs, Hitchin, UK). Resulting libraries were purified with AMPure XP beads (Agencourt, Beckman Coulter, High Wycombe, UK) and quantified using fluorometry (Qubit, Thermo Fisher Scientific. Loughborough, UK). We selected the poly(A) enrichment method as, although it is biased toward favouring the amplification of polyadenylated RNA viruses (compared to viruses without a poly-A tail) and correspondingly leads to biased coverage in increased depth toward the 3′ end, the majority of our viruses of interest are polyadenylated so we felt the amplification bias would overall increase our ability to detect these viruses, DWV particularly. Negative controls comprised two sequenced libraries prepared alongside the samples but with no template and two previously generated *E. coli* libraries. Libraries were randomly assigned one of eight lanes (pooling strategy is shown in Table S2) and RNAseq was carried out on a HiSeq 4000 (Illumina, Cambridge, UK) at The Centre for Genomic Research (CGR) at the University of Liverpool (UK) generating 150 bp paired end sequences.

### 2.4. Data Quality Control

Initial quality control of generated RNAseq data (QC) was carried out at CGR by using Cutadapt (v1.2.1, Dortmund, Germany) to remove adapters, followed by Sickle (v1.200, Davis, CA, USA) using a minimum window quality score of 20 to further trim poor quality reads. Only reads which passed QC for both R1 and R2 were kept for subsequent analysis (singlet R0 reads were discarded).

### 2.5. Identification and Quantification of DWV-Like Reads and Identification of Recombinants

Reads from each sample which passed QC (R1 reads in fasta format) were imported into Geneious v10.1.3 (Biomatters, Aukland, New Zealand) and the “map to reference” tool was used to competitively map the raw reads to the DWV-A, B, and C genomes (accession numbers NC004830.2, NC_006494.1 and CEND0100000.1, respectively). To avoid falsely elevated read counts, reads giving multiple best matches were discarded and the alignments were produced mapping each read once only and with no fine tuning. For each sample, read counts mapping to each variant were firstly determined separately, then DWV-A, -B, and -C reads were added together to give total DWV reads which were then pooled by taxa and according to whether they originated from a *Varroa*, or no *Varroa* island. All read counts were expressed as reads per kilobase million (RPKM). Statistical differences were calculated in RStudio (version 1.2.5042, Boston, MA, USA), using Kruskal–Wallis tests as data were not normally distributed. Plots were created using ggplot2 (version 3.3.0) with ggpubr (version 0.4.0), forcats (version 0.5.0) and dplyr (version 0.8.5) packages in R studio (version 1.2.5042).

Coverage plots were then produced for all samples to confirm coverage across the genomes and to identify recombination break points, this was done for each honey bee sample separately (each having been prepared using pools of five individuals) and pooled for all other samples according to whether they were from an island with or without *Varroa*.

### 2.6. Determination of the Extent to Which Common DWV Variants Are Shared

For each sample, consensus DWV-A and -B contigs were generated from the Geneious alignments (there were insufficient DWV-C reads for analysis). Muscle alignments were then carried out on 507 bp fragments of the RdRp gene on DWV-A and -B reference genomes (positions 8016–8522 and 7989–8495, respectively) and sequences with stretches of ambiguous bases removed. IQ Tree v1.6.1 (Canberra, Australia [23]) was then used to create a maximum likelihood phylogeny using 100 bootstraps after using ModelFinder (Canberra, Australia [24]) to determine the appropriate model according to Bayesian Information Criterion (BIC) scoring. Editing was carried out using FigTree v1.4.4 (Edinburgh, UK [25]).

### 2.7. Determination of DWV Quasispecies Diversity

DWV quasispecies diversity was calculated as the number of variable bases present across a 2000 bp region of the non-structural block (7000–9000 bp on the reference genome). We did not assess the whole genome because the majority of samples did not show full length DWV coverage. This is a result of the combination of overall low levels of DWV in many samples and the bias of the poly(A) enrichment method of library preparation toward sequencing the 3′ end of the genomes. Variable bases were identified in the DWV-A and -B alignments (prepared earlier by competitive mapping to each master variant) by using the Geneious “Find variations/SNPs” feature, with a minimum coverage of 10 reads, minimum variant frequency of 0.2, assuming a quality score of 20 and ignoring the reference sequence. Again, honey bee samples were analysed individually, and other samples were grouped to provide sufficient coverage. There were insufficient DWV-C reads for analysis.

### 2.8. Identification of Other Honey Bee-Associated Viruses

To understand whether the presence of *Varroa* had had an effect on the prevalence or abundance of other honey bee-associated viruses, in honey bees or other insects, we used BLASTn with an e-value of 10^−5^ against a database containing 14 honey bee-associated virus genomes [26] as well reference genomes for Moku and Milolii viruses; both of which are known to be present in Hawaiian insect populations [27,28]. Accession numbers for all virus reference genomes are given in Table S3. Read counts were expressed as reads per kilobase million (RPKM) and were used to generate heatmaps in Rstudio (version 1.2.5042) using ggplot2 (version 3.3.0). We ran Generalized Linear Models (GLM) using the “*lme4*” R package [29] with control-corrected RPKM values as the response variable and virus, *Varroa* status and taxon as fixed effects. We performed ANOVAs on the models using the “*car*” R package [30] and identified where results differed significantly using Post-hoc Tukey tests using the “*lsmeans*” R package [31].

## 3. Results

### 3.1. Sequence Data Statistics and Quality Control

From 127 samples we sequenced a median of 14,438,289 reads after QC (range = 1,589,666–33,823,756). One potter wasp sample was removed from analysis due to poor quality. The median sampling depth from the control samples was 4,289,666. Sampling depths for individual samples are given in Table S2. Whilst we did detect some bee-associated virus reads in the two water controls, suggesting some level of contamination either through barcode switching or in the laboratory, the six *E. coli* libraries contained a much lower proportion of contaminant reads (Figure S1, Table S2). The highest abundance of any bee-associated virus reads in an *E. coli* library was 5.71 RPKM; therefore, all virus detections at lower abundance than this were discarded. All sequence data files are publicly available in the NCBI Sequence Read Archive (SRA) under BioProject ID PRJNA670741.

### 3.2. Is Varroa-Presence Associated with an Increase in DWV in Diverse Taxa?

**Figure 1:**
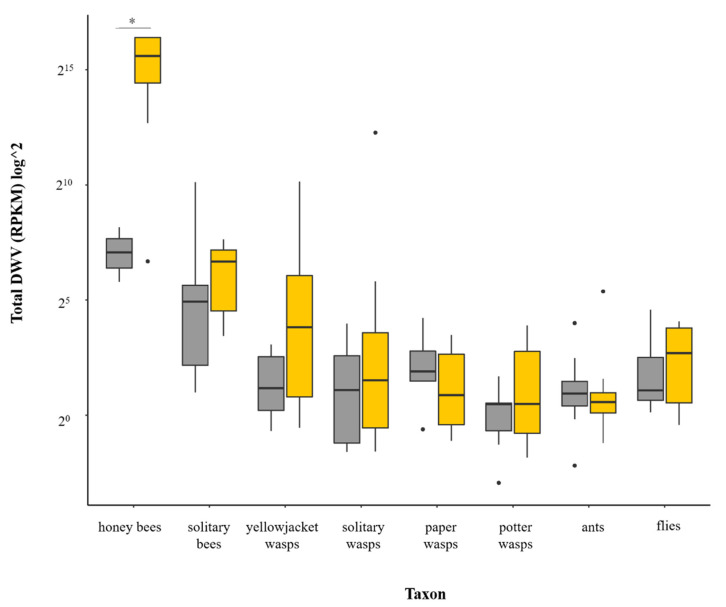
DWV read counts detected in each taxon group. Averages were calculated for each taxon with individuals pooled according to whether they were collected from an island with no-*Varroa* (grey) or with *Varroa* (yellow). Boxes represent 25th to 75th percentiles with medians shown with a black bar, estimated confidence intervals shown with hinges and outliers represented by dots. Statistical differences between *Varroa* and no *Varroa* samples in each taxon group (Kruskal Wallis tests) are denoted by an asterisk.

Comparing the averaged total DWV read counts within each taxon group revealed that, whilst the honey bee samples were strongly affected by the presence of *Varroa* in the population (*X^2^* = 8.0404, *df* = 1, *p* = <0.005), no significant differences were seen in any other insect group (Figure 1). Rather, the vast majority of samples of all other taxa contained a low number of DWV reads, comparable to the amount detected in honey bees from *Varroa*-free islands, where DWV loads are typically very low. This was, however, variable between individual samples, for all taxa. No clear effect was seen between honey bees collected from the apiary (“apiary” sites) and those collected from areas away from managed bees (“non-apiary” sites), either from the islands with *Varroa* (*X^2^* = 0.083, *df* = 1, *p* = 0.772), or without (*X^2^* = 1.00, *df* = 1, *p* = 0.317). Thus, samples were pooled according to whether they had been collected from *Varroa* or *Varroa*-free islands regardless of at which sites they had been collected (“apiary” or “non-apiary” sites) (Figure 1).

### 3.3. Are Particular DWV Variants or Recombinants Thereof Correlated with Host Taxon or Varroa Presence?

**Figure 2:**
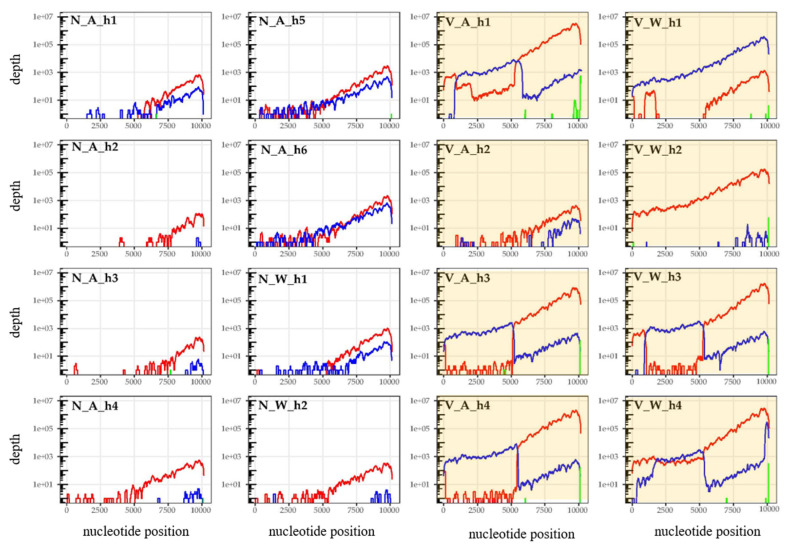
DWV genome coverage plots for all honey bee samples. Samples collected from *Varroa*-free islands are shown with white backgrounds (sample names beginning “N”) and those from islands with *Varroa* are shown with yellow (sample names beginning “V”). Those collected from apiary sites (“apiary”) are shown with an “A” and those collected away from managed bees (“non-apiary”) are shown with a “W”. DWV-A coverage is shown in red, DWV-B in blue and DWV-C in green (negligible amounts).

DWV-A was by far the most common master variant in this study, accounting for 87% of all DWV reads (RPKM). DWV-B reads were seen in a number of samples (belonging to all taxa and regardless of *Varroa* status), generally at lower levels. Where DWV-B was most abundant as a recombinant with DWV-A in honey bee samples with high levels of total DWV, from islands with *Varroa* (Figure 2, Figure S2 for plots displaying maximized y axes for each plot). DWV-C reads were rarely seen in this study. All plots show the characteristic coverage pattern generated using oligo d(T) enriched RNAseq libraries.

**Figure 3:**
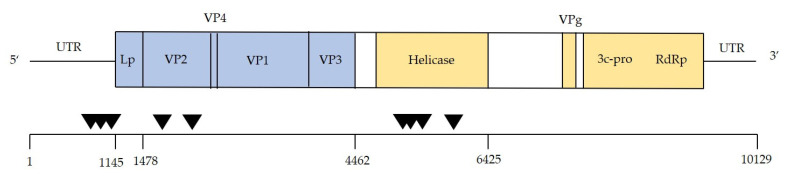
Schematic of the DWV genome showing approximate recombination breakpoints seen in this study denoted by a black triangle. Structural genes are shown in blue and non-structural genes in yellow. Nucleotide positions are shown according the DWV-A genome.

**Figure 4:**
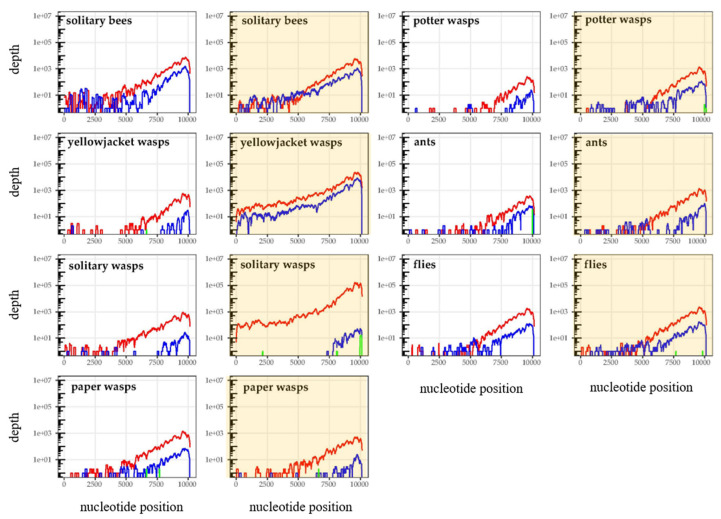
DWV coverage plots for all non-honey bee samples, pooled according to whether they were collected from with-*Varroa* (yellow background) or no-*Varroa* (white background) islands. DWV-A coverage is shown in red, DWV-B in blue and DWV-C in green. Each plot shows the total mapped DWV reads from the four individual samples per group.

For the honey bees, the taxon which showed by far the highest amount of DWV, competitive alignment plots were produced for each sample (which comprised five pooled individuals) separately. Diverse DWV-A and -B recombinants could be seen dominating the majority of honey bee samples collected from islands where *Varroa* was present, as evidenced by the sharp switching of dominant genotypes at various points across the genome (Figure 2). Recombination breakpoints were commonly seen in the Helicase gene (breakpoints ~5000–5500 bp) and in the 5′ UTR, with breakpoints in the VP2 region also present, but not dominating in samples (summarized in Figure 3). However, this was variable between samples and no consistent differences were seen when comparing apiary samples to feral bees. For all other taxa, samples were pooled by taxa and *Varroa* status, to increase coverage depth and resolution of potential recombinants and while no obvious recombinants were seen, the low read depths were insufficient to confirm their absence (Figure 4, Figure S3). Indeed, the only groups that showed full length DWV coverage were the yellowjacket wasps and the solitary wasps, from the islands with *Varroa*. Again, all plots show the expected coverage pattern across the genome.

### 3.4. Are Common DWV Variants Found to Dominate Across Taxa?

**Figure 5:**
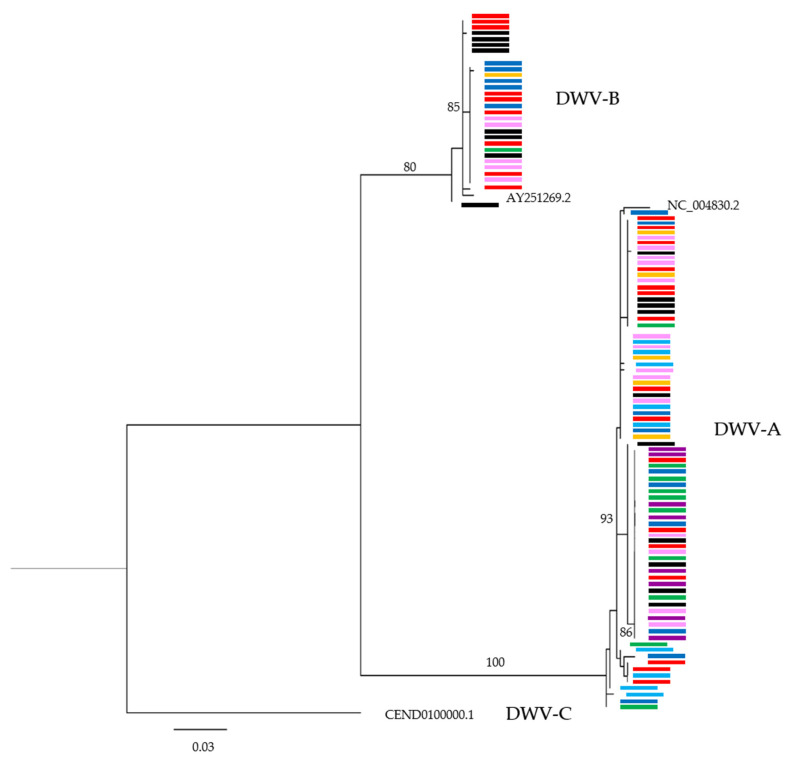
Maximum likelihood phylogeny of a 507 bp RdRp fragment of the DWV genome. Coloured bars donate sequences obtained from each taxon group. Sequences from honey bee samples are shown in black, solitary bees in red, solitary wasps in magenta, yellowjacket wasps in blue, potter wasps in orange, paper wasps in green, ants in light blue and flies in pale pink. Bootstrap support values of >80% are shown.

A maximum likelihood phylogeny was created using consensus DWV-A and -B sequences for each sample containing reads spanning the 507 bp RdRp fragment of interest, using an HKY + F + G4 model, with 100 bootstraps. This revealed no apparent clustering within in the DWV-A or DWV-B clades, either by taxon or *Varroa* status (Figure 5). Rather, very similar DWV-A and DWV-B sequences were shared amongst all samples, with the differences that were seen having generally low support.

### 3.5. Has the Establishment of Varroa Caused a Decrease in DWV Quasispecies Diversity in Non-Apis Species?

**Figure 6:**
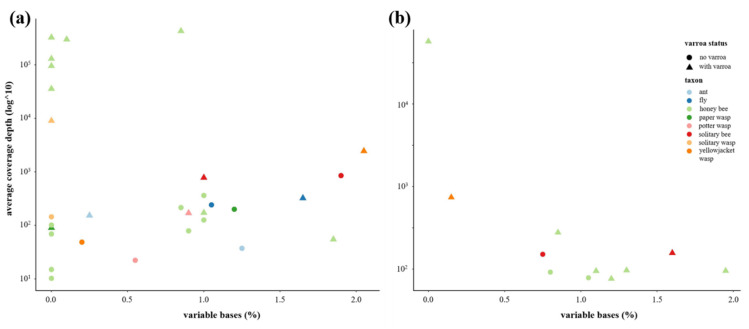
DWV diversity as represented by the proportion of variable base positions (%) between 7000–9000 bp on the reference genomes, as a function of average read depth across the same fragment for (**a**) DWV-A; and (**b**) DWV-B.

The DWV diversity, as indicated by the proportion of variable bases in a 2000 bp fragment within the non-structural block (spanning the genome linked viral protein (VPg), 3C protease and RNA-dependent RNA polymerase (RdRp) genes), did not show any consistent pattern when comparing samples from islands with and without *Varroa* (Figure 6). A trend was seen, however, with respect to read depth; while the samples with low read depth were highly variable in their diversity, the samples with the highest depths (generally, the honey bee/with *Varroa* samples) showed consistently low diversity. Analysis of DWV-B was restricted to only the subset of samples (mostly honey bees from the “with *Varroa*” group) that contained sufficient coverage across the region of interest. As the bias of the poly(A) enrichment method of library preparation for RNAseq meant that many of our low-level DWV samples contained insufficient DWV coverage depth at the 5′ end to analyse whole genomes, there was insufficient DWV-C data for analysis.

### 3.6. Has the Establishment of Varroa Caused a Shift in the Viral Landscapes of Insects of Different Taxa?

**Figure 7:**
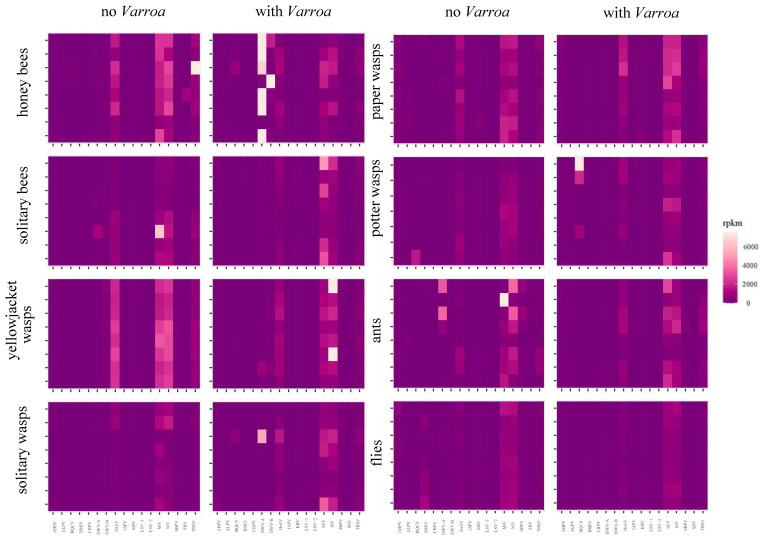
Heatmap showing the amounts of bee virus-associated reads (RPKM) in each individual sample, presented grouping each taxon and further separating by *Varroa* status (left panels no *Varroa*, right panels with *Varroa*).

**Figure 8:**
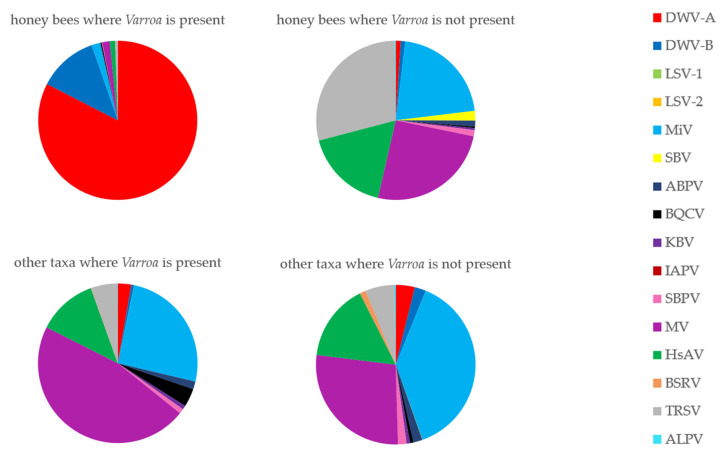
Pie charts showing compositions of the honey bee-associated viromes of honey bees and all other taxa sampled combined, from islands with and without *Varroa*.

With the exception of DWV in the honey bees belonging to the “with *Varroa”* group, generally, all viruses in this study were detected at low levels across all taxa (Figure 7). Moku virus (MV), Milolii virus (MiV) and Halictus scabiosae Adlicon virus (HsAV) were the more abundant viruses throughout the data, but no clear pattern was seen comparing either taxon groups or *Varroa* status (with and no *Varroa*). Overall, there were significant effects on viral abundance of *Varroa* status, taxon and virus (Figure S4), however when comparing effect sizes across all possible three-way interactions, statistically significant differences were only seen in the honey bees/DWV-A/with *Varroa* group, and to a lesser degree, the honey bees/DWV-B/with *Varroa*, yellowjacket wasps/MV/with *Varroa* and ants/MiV/no *Varroa* groups (Figure S4). Furthermore, when honey bee data were excluded from the model, there was no significant effect of *Varroa* status, neither by itself, nor in interactions with other factors. There were occasional samples with high levels of particular viruses—e.g., a potter wasp from the no *Varroa* group contained high levels of black queen cell virus (BQCV) and a honey bee from the no *Varroa* group contained high levels of tobacco ringspot virus (TRSV)—but these instances were rare in the data and overall the composition of the honey bee-associated RNA viromes of the non-honey bee samples were comparable from islands with and without *Varroa* (Figure 8).

## 4. Discussion

The results of this study confirm previous findings; that the introduction of *Varroa* to a European honey bee population results in shifting the honey bee virome to becoming dominated by DWV [4,11,32]. However, our data suggest that, after 5 years exposure to *Varroa*] this effect is limited to honey bees, with no change to DWV levels observed in any other insect group. Whilst other taxa contained similar DWV variants, as were present in the honey bees, the levels were consistently very low regardless of where they were detected (islands with or without *Varroa*), at levels comparable with those in the honey bees collected from islands without *Varroa*.

Studies have shown that DWV-B is now the prevailing master variant in England [9], France [11] and is increasing in mainland USA [9,33]. In some countries, such as South Africa [34], DWV-B remains the dominant master variant. However, our data show that, in Hawaii, DWV-A is still the dominant master variant, although DWV-B is also present and could potentially be increasing, indicated by its increased prevalence compared to our previous work [4]. Interestingly, contrary to other studies [9,11,33], our samples were frequently co-infected with both master variants (Figure 2 and Figure 3), potentially supporting the findings of Ryabov et al. [35] that the different master variants are adapted to co-exist. While a recent experimental co-infection study showed that DWV-B replicated to higher levels than DWV-A in co-infections [36], and the vast majority of our samples contained more DWV-A reads than DWV-B, suggesting a lack of any strong competitive exclusion in this population. This was a similar finding as in the experimental infection study by Tehel et al. [37] in which DWV-A replicated to slightly higher levels than DWV-B when co-infected. As most samples contained insufficient DWV-B reads for whole genome comparisons, phylogenetic analysis of consensus DWV-A RdRp sequences showed the viral genotypes harboured to be similar across taxa and regardless of where they were collected. This may be explained by those variants which were present pre-*Varroa* introduction and have remained present and continue to circulate at low levels in diverse hosts, or alternatively, by the DWV detected in other insects that reflects the genotypes spilling over from the honey bees at the time.

Perhaps unsurprisingly, given the frequency of co-infections, DWV-A and -B recombinants were also prevalent in our honey bee samples. Broadly, the recombination breakpoints were similar to those seen in other studies using honey bees exposed to *Varroa* and DWV [38,39]; recombinant genomes comprised DWV-B structural genes and DWV-A non-structural genes, with some also containing a DWV-A 5′ untranslated region as in studies by [33,40], although break points were variable. Present recombinants usually dominated, suggesting these harbour a combination of traits of both master variants well suited to replicating to high levels in the honey bee host. Interestingly, some honey bee samples from islands with *Varroa* (one sample from an “apiary” site, V_A_h1 and two samples from “non-apiary” sites, V_W_h1 and V_W_h4), appeared to show a combination of different recombinants and full length genomes, suggesting a dynamic DWV population with multiple variants and recombinants with high replication rates, although this may be a result of using pooled bees. We did not detect any DWV-A and B recombinants in the honey bee samples from islands with no *Varroa* nor any other insect. While we cannot eliminate the possibility of their presence at low levels, we did see full length genome coverage for two sample groups; the yellowjacket wasps (DWV-A and DWV-B) and the solitary wasps (DWV-A only), both from islands with *Varroa*. A potential absence of recombinants from other insects may be a result of the benefits or detriments of recombinant forms being specific to the honey bee host. The data in the present study differ to our previous findings [17], where we saw a number of non-honey bee samples containing high DWV levels and recombinants. We suggest this may be a result of different collection methodologies. Previously we collected apiary pests from inside or adjacent to hives where they were frequently interacting with DWV-infected honey bees as well as potentially contaminated hive products. Thus, those insects may have experienced a higher degree of DWV exposure compared to those in this study which were collected more broadly, generally from gardens and roadsides. Furthermore, our previous study involved pre-screening for DWV presence prior to library preparation where this study did not.

As previously demonstrated by both [4,5], honey bee samples with the highest amounts of DWV generally showed low viral diversity and those with lower DWV read depth were generally more diverse, although the diversity of the low read depth samples was highly variable and some had no variable bases. Studies have suggested, following *Varroa* introduction, that DWV undergoes a bottleneck following the introduction of the new transmission route and subsequent rapid expansion of genotypes (both DWV-A and B) [11,35]. The pattern of low level, generally more diverse DWV was also seen in the other insect groups. Interestingly, however, there were no consistent differences between those samples collected from islands with *Varroa* to those without. This is contrary to the findings of Loope et al. [18], who found comparable prevalence but a reduction in genetic diversity in the yellowjacket wasp (*V. pensylvanica*) following *Varroa* introduction to the Big Island (Hawaii). In fact, the yellowjacket wasp samples in this study showed more DWV-A diversity when *Varroa* was present. The diversity of the DWV-B population was low in the “with *Varroa*” samples, but there were insufficient DWV-B data from “no *Varroa*” islands for comparison. We hypothesize that the reason for the difference may again be due to the sampling difference; the study by Loope et al. [18] used wasps which were actively preying upon honey bees. As such, it may be that those individuals living near, or predominantly foraging on honey bees experience DWV infections much more closely linked to the honey bees, compared to individuals which forage elsewhere and presumably may have a more varied diet including a smaller fraction of (DWV-infected) honey bees. The results here are also strikingly different to the findings of Santamaria et al. [14], who found a dramatic increase in DWV prevalence in paper wasps and a solitary bee on Hawaiian Islands with *Varroa*. While we cannot explain this difference in the results, that study used RT-PCR for prevalence information only (i.e., no quantification) and as such it may be that their positive detections were of low viral loads and so differences were seen, but impacts may be low.

The lack of high titre, low diversity DWV samples in other insects suggests that no variants dominate in non-honey bee hosts, with perhaps the exception of one solitary wasp sample from the “with *Varroa”* group. Furthermore, this, along with the general DWV variant similarity amongst samples means we cannot determine whether the samples were experiencing active infections. While the replication of DWV genotypes has been detected in other hosts, such as *Varroa* [41,42], ants (Formicidae) [43], stingless bees (Apidae) [44], solitary bees (Colletidae [44], Andrenidae [13] and Megachilidae [45]) and hornets (Vespidae) [46], and DWV has been detected in symptomatic (deformed) bumblebees (*Bombus spp*.) [47] and hornets (*Vespa velutina*) [48], conclusive studies on the ability of DWV to cause pathology in non-honey bee hosts have largely been limited to bumblebees [15,19]. Another recent study, however, found no effect on mortality in experimentally infected bumblebees [49], suggesting that DWV infection is not uniformly virulent in bumblebees. As such, DWV is clearly well-adapted and has a diverse and wide insect host range. The host–virus interaction appears to be in steady sate (asymptomatic or persistent), with virulence and overt infections uncommon in nature, only under certain conditions—i.e., when *Varroa* jumped to the European honey bee. Furthermore, experimental inoculations of non-*Apis* bees have required high titres for oral infection of bumblebees [20,49], suggesting infections under field conditions transmission resulting in active infection would be unlikely. Furthermore, Dolezal et al. [50] also saw no evidence of viral replication or elevated mortality in wild bees (*Megachile rotundata* and *Colletes inequalis*) fed high doses of a DWV, Israeli acute paralysis virus (IAPV) and Sacbrood virus (SBV) inoculum.

Considering the other (non DWV) honey bee-associated viruses investigated in this study, the vast majority of samples showed a similar pattern of abundance. Virus reads were generally present at low amounts regardless of taxa or *Varroa*-status, with Moku (MV), Milolii (MiV) and Halictus scabiosae Adlicon virus (HsAV) being the most abundant. Loope et al. [18] suggested that *Varroa* may have indirectly altered the viromes (of yellowjacket wasps), perhaps through virus–virus or immunity mechanisms in different hosts, but we do not see any evidence of that in our data. In fact, even the honey bees which were heavily dominated by DWV showed comparable levels of other viruses with the other samples, suggesting an absence of any strong virus–virus interactions. The low-level detection of many of these viruses throughout our data, however, do suggest some level of consistent intra-host transmission is occurring, although in the majority of cases the low numbers of reads means false positives cannot be ruled out. The fact that viruses are present, which show similar patterns amongst hosts, suggests that, if an event such as the introduction of a novel transmission route occurs for one of these viruses, there may be significant risk to diverse hosts. This is especially concerning as at least BQCV, SBV and IAPV are known to also replicate in diverse hosts [49,50,51] and we still know relatively little about three of the more abundant viruses in this study (MV, MiV, HsAV). Another unusual finding of this study was the notable absence of BQCV and SBV in the honey bee population, a contrast to the majority of studies [52,53]. These two viruses are often also found in wild bees from the same geographic regions [12,54]—as such, the lack of them in the non-honey bees in Hawaii supports the hypothesis that their presence in honey bees in other regions is driving their abundance in wild bees. Interestingly, however, there were BQCV reads in some wasp samples; in particular, one potter wasp’s BQCV read count was similar to those levels of DWV in honey bees. This suggests BQCV is able to replicate in at least some wasp species and as such may be a threat. Although, as with DWV, it must be noted that replication does equate to pathology.

While we did not find any evidence of indirect effects of *Varroa* on wild insects, it is likely that honey bees generally have an effect on viromes in wild insects. Fung [55] showed an absence of common honey bee-associated viruses in wild bees where honey bees are absent from the environment (due to altitude or aridity) implying, unsurprisingly, that honey bees are the source of these viruses. In congruence with our data, the study also showed no difference whether the co-occurring honey bees were managed or not. Given that honey bees are near ubiquitous in Hawaii and have been present since 1857 [56], we cannot confirm the original source of the viruses detected in this study nor the directionality of transmission.

## 5. Conclusions

In conclusion, whilst the introduction of *Varroa* to Hawaii (by extension to the Western world) has had a strong effect on honey bees by causing a dramatic increase in low-diversity DWV, the effect appears to be largely restricted to honey bees, with no corresponding increase in DWV in other insects. This is particularly true if the frequency of direct interactions with infected honey bees is low. Furthermore, no changes were seen to the abundance of other honey bee-associated viruses, in honey bees or other taxa. While this on one hand may be taken as alleviating some concerns about the indirect effects of *Varroa*, we cannot discount the possibility of the absence of high amounts of DWV in other taxa being a result of highly virulent infections killing the infected hosts and thus not being captured by this survey. Furthermore, our relatively limited sampling (eight replicates per taxon, per group) means our ability to detect prevalence in individual taxa is limited. Our data suggest that the impact on wild insects of high levels of DWV in honey bees may be less than previously thought, with bumblebees (which are not present in Hawaii) perhaps being the exception [11,15], and while we have seen that DWV can and does spillover into diverse taxa, rarely does this result in high level pathological effects in wild insects.

## Supplementary Materials

The following are available online at https://www.mdpi.com/1999-4915/12/11/1229/s1, Figure S1. Heatmap showing the amounts of bee virus-associated reads (RPKM) in each individual control sample. Six controls comprised of *E. coli* libraries are denoted “Nextlib” and two water controls are denoted “control_ex”. Figure S2. DWV genome coverage plots for all honey bee samples. Samples collected from *Varroa*-free islands are shown with white backgrounds (sample names beginning “N”) and those from islands with *Varroa* are shown with yellow (sample names beginning “V”). Those collected from apiary sites (“apiary”) are shown with an “A” and those collected away from managed bees (“non-apiary”) are shown with a “W”. DWV-A coverage is shown in red, DWV-B in blue and DWV-C in green (negligible amounts). Y axis limits differ between plots according to coverage depths. Figure S3: DWV coverage plots for all non-honey bee samples, pooled according to whether they were collected from with-*Varroa* (yellow background) or *Varroa*-free (white background) islands. DWV-A coverage is shown in red, DWV-B in blue and DWV-C in green. Each plot shows the total mapped DWV reads from the four individual samples per group. Y axis limits differ between plots according to coverage depths. Figure S4: Effect sizes of interactions between virus, *Varroa* status and taxon, calculated as least square means on the results of a Generalised Linear Model. Effect sizes are considered significant when 95% confidence intervals do not span zero. Table S1: Additional information for all samples in this study. Table S2: RNAseq library information for individual samples presented according to pooling strategy. Table S3: All honey bee-associated viruses tested for in this study.
