# Supplementary material for: RNAseq of Deformed Wing Virus and Other Honey Bee-Associated Viruses in Eight Insect Taxa with or without Varroa Infestation
Source: Viruses. 2020 Oct 29;12(11):1229. doi: 10.3390/v12111229 (PMC7692275; doi:10.3390/v12111229)
Supplement: Supplementary file 1 [file viruses-12-01229-s001.zip › Supplementary_v2/Supp_fg_s1_v2.docx]

**Supplementary materials**


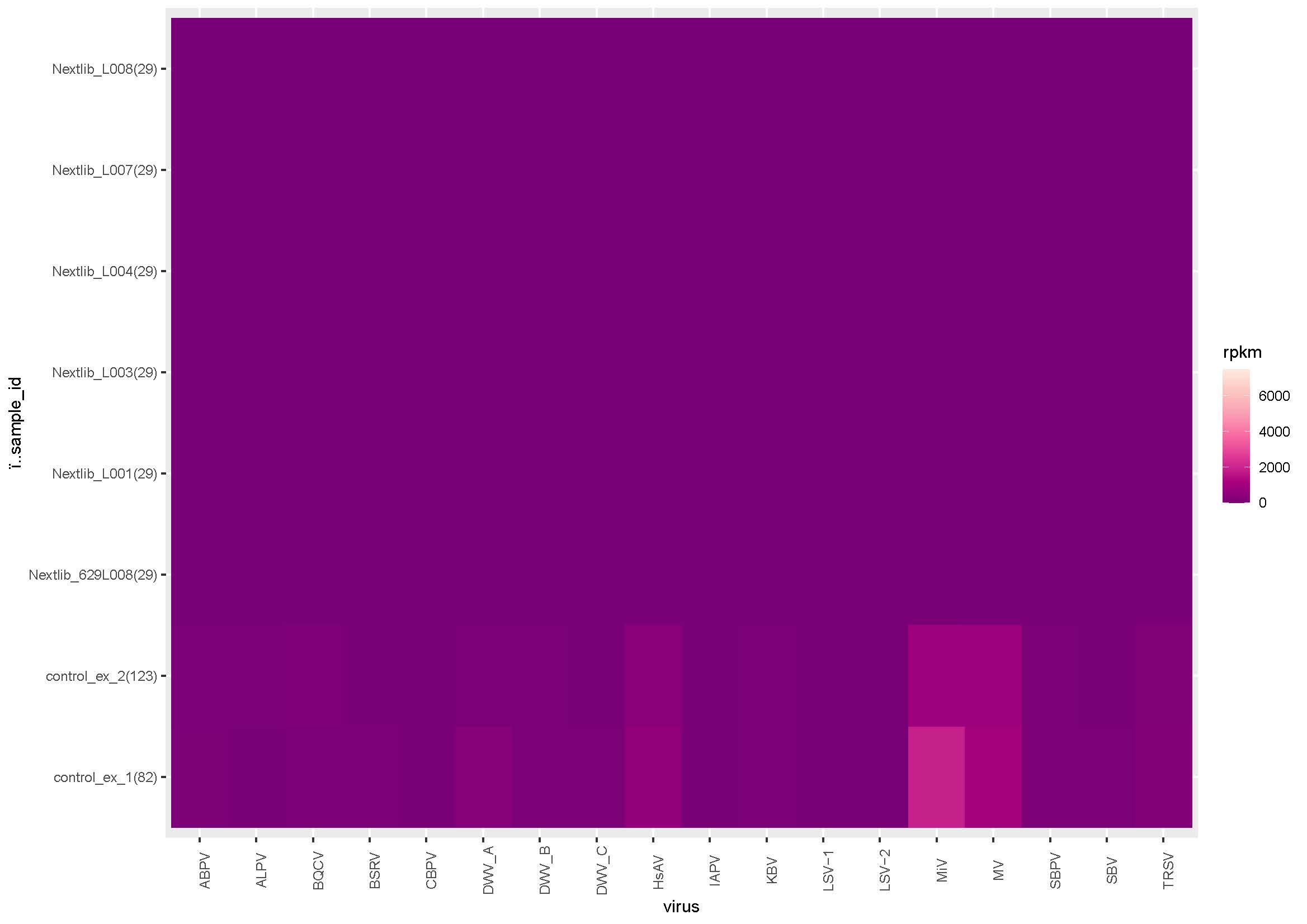


**Supplementary Figure S1**: Heatmap showing the amounts of bee virus-associated reads (RPKM) in each individual control sample. Six controls comprised of *E.coli* libraries are denoted “Nextlib” and two water controls are denoted “control_ex”.
