# Supplementary material for: RNAseq of Deformed Wing Virus and Other Honey Bee-Associated Viruses in Eight Insect Taxa with or without Varroa Infestation
Source: Viruses. 2020 Oct 29;12(11):1229. doi: 10.3390/v12111229 (PMC7692275; doi:10.3390/v12111229)
Supplement: Supplementary file 1 [file viruses-12-01229-s001.zip › Supplementary_v2/Supp_fg_s2_v2.docx]

**
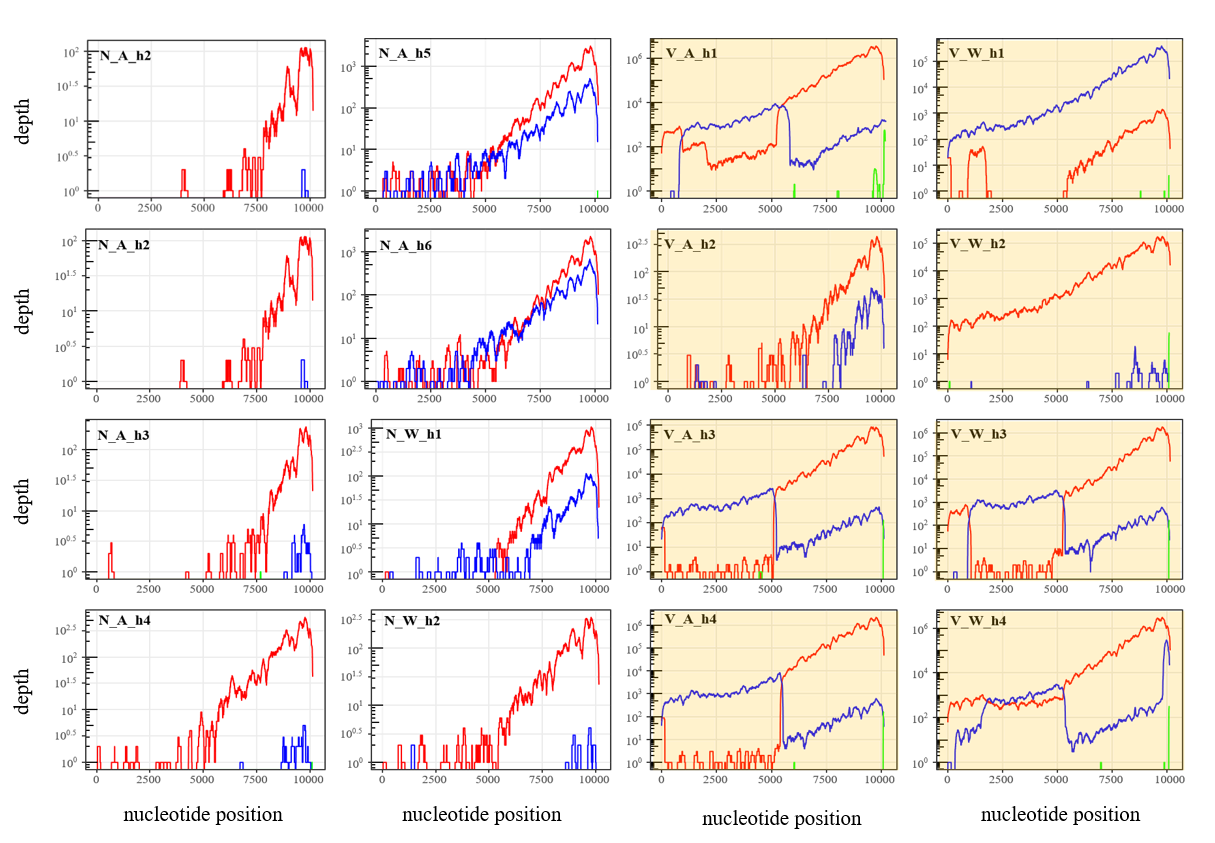
**

**Supplementary Figure S2**. DWV genome coverage plots for all honey bee samples. Samples collected from *Varroa*-free islands are shown with white backgrounds (sample names beginning ‘N’) and those from islands with *Varroa* are shown with yellow (sample names beginning ‘V’). Those collected from apiary sites (‘apiary’) are shown an ‘A’ and those collected away from managed bees (‘non-apiary’) are shown with a ‘W’. DWV-A coverage is shown in red, DWV-B in blue and DWV-C in green (negligible amounts). Y axis limits differ between plots according to coverage depths.
