# Supplementary material for: RNAseq of Deformed Wing Virus and Other Honey Bee-Associated Viruses in Eight Insect Taxa with or without Varroa Infestation
Source: Viruses. 2020 Oct 29;12(11):1229. doi: 10.3390/v12111229 (PMC7692275; doi:10.3390/v12111229)
Supplement: Supplementary file 1 [file viruses-12-01229-s001.zip › Supplementary_v2/Supp_fg_s3_v2.docx]

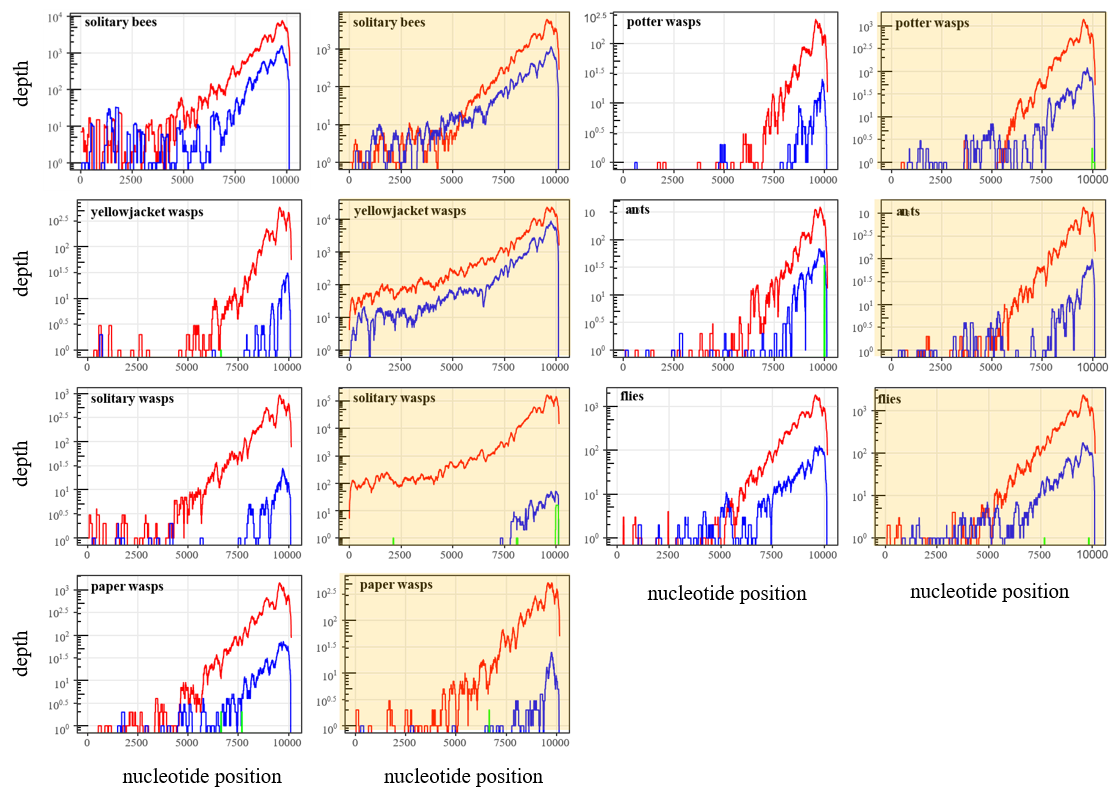


**Supplementary Figure S3**. DWV coverage plots for all non-honey bee samples, pooled according to whether they were collected from with-*Varroa* (yellow background) or *Varroa*-free (white background) islands. DWV-A coverage is shown in red, DWV-B in blue and DWV-C in green. Each plot shows the total mapped DWV reads from the four individual samples per group. Y axis limits differ between plots according to coverage depths.
