# Supplementary material for: RNAseq of Deformed Wing Virus and Other Honey Bee-Associated Viruses in Eight Insect Taxa with or without Varroa Infestation
Source: Viruses. 2020 Oct 29;12(11):1229. doi: 10.3390/v12111229 (PMC7692275; doi:10.3390/v12111229)
Supplement: Supplementary file 1 [file viruses-12-01229-s001.zip › Supplementary_v2/Supp_fg_s4_v2.docx]

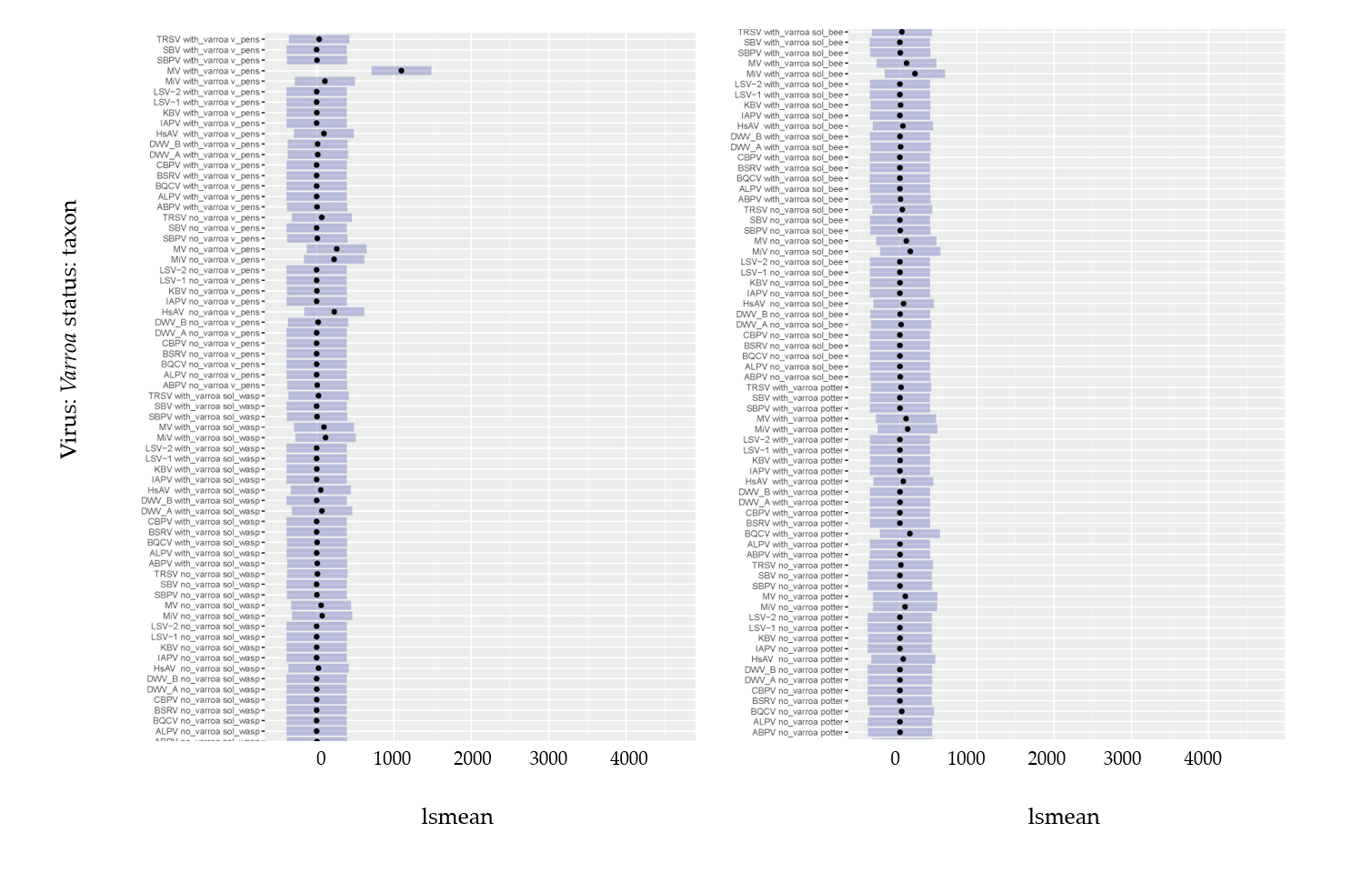


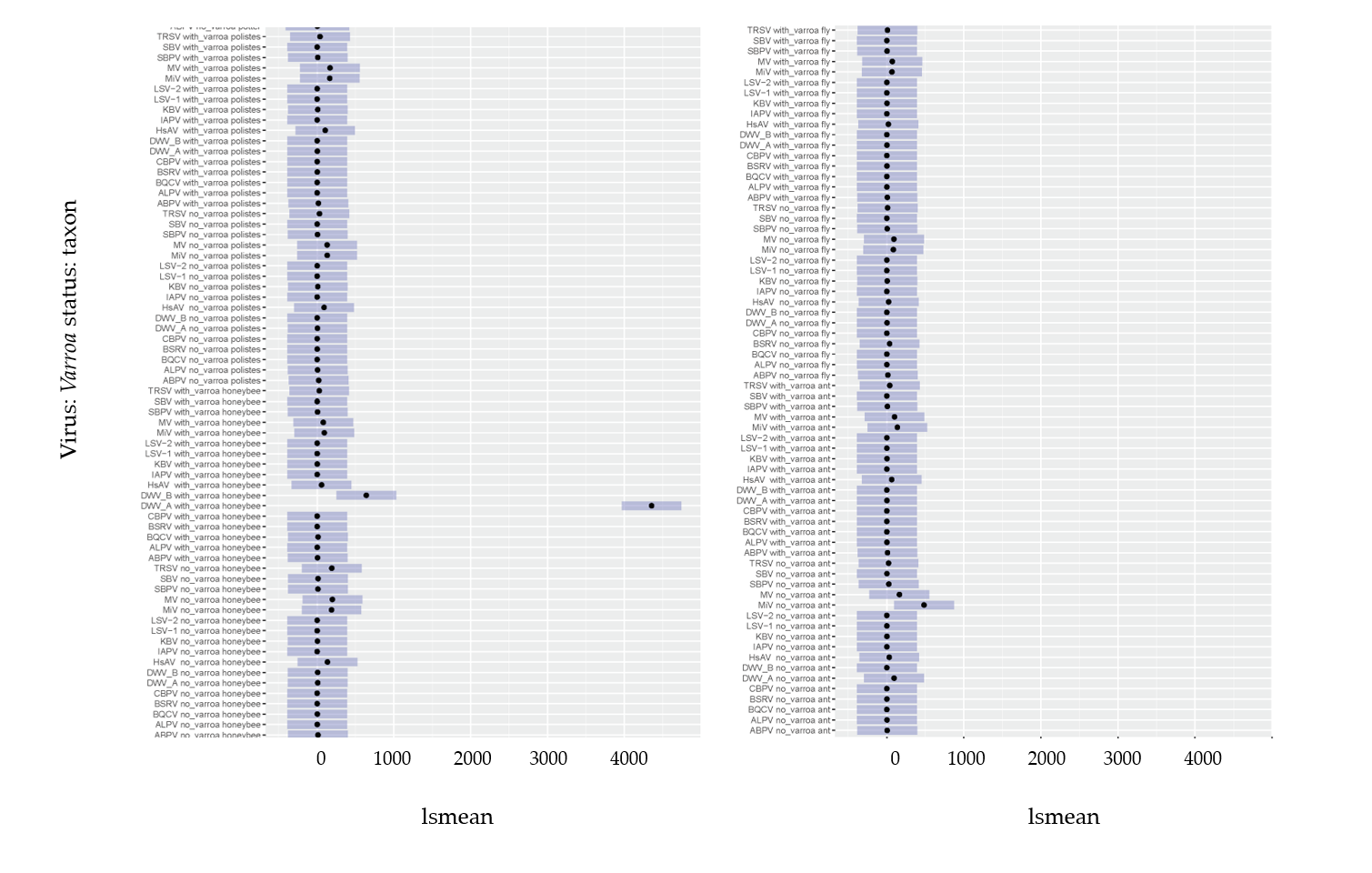


**Supplementary Figure S4.**Effect sizes of interactions between virus, *Varroa* status and taxon, calculated as least square means on the results of a Generalised Linear Model. Effect sizes are considered significant when 95% confidence intervals do not span zero.
