# Supplementary material for: RNAseq of Deformed Wing Virus and Other Honey Bee-Associated Viruses in Eight Insect Taxa with or without Varroa Infestation
Source: Viruses. 2020 Oct 29;12(11):1229. doi: 10.3390/v12111229 (PMC7692275; doi:10.3390/v12111229)
Supplement: Supplementary file 1 [file viruses-12-01229-s001.zip › Supplementary_v2/Supp_table_s1_v2.docx]

**Supplementary Table S1**. Additional information for all samples in this study.

| **sample id** | **taxon group** | **v_status** | **site** | **original name** | **island** | **location** | **morphological ID** | **amount used for RNA extraction** | **collection date** |
| --- | --- | --- | --- | --- | --- | --- | --- | --- | --- |
| V_A_h1(1) | honeybees | w_varroa | apiary | H15_Ow1a | Oahu | Waimanalo | *Apis mellifera* | 30mg (5 adults) | 24/11/2015 |
| V_A_h2(62) | honeybees | w_varroa | apiary | H15_Bg6 | Big Island | Garnett | *Apis mellifera* | 30mg (5 adults) | 21/11/2015 |
| V_A_h3(124) | honeybees | w_varroa | apiary | H15_Opa1 | Oahu | Howard - Party | *Apis mellifera* | 30mg (5 adults) | 24/11/2015 |
| V_A_h4(125) | honeybees | w_varroa | apiary | H15_OPat2 | Oahu | Pat - UH | Apis mellifera | 30mg (5 adults) | 17/10/2016 |
| V_W_h1(2) | honeybees | w_varroa | non-apiray | H15_Ba1a | Big Island | Amy Greenwell Botanical Garden | *Apis mellifera* | 30mg (5 adults) | 21/11/2015 |
| V_W_h2(63) | honeybees | w_varroa | non-apiray | H15_Oc3 | Oahu | Coco head | *Apis mellifera* | 30mg (5 adults) | 17/11/2015 |
| V_W_h3(126) | honeybees | w_varroa | non-apiray | H16_OWM1 | Oahu | Waahila Ridge Manoa | *Apis mellifera* | 30mg (5 adults) | 7/04/2016 |
| V_W_h4(127) | honeybees | w_varroa | non-apiray | H16_ORr1 | Oahu | Ridge road | *Apis mellifera* | 30mg (5 adults) | 7/04/2016 |
| N_A_h1(3) | honeybees | no_varroa | apiary | H15_Mb1a | Molokai | Brenda | *Apis mellifera* | 30mg (5 adults) | 19/11/2015 |
| N_A_h2(42) | honeybees | no_varroa | apiary | H15_Mb2a | Molokai | Brenda | *Apis mellifera* | 30mg (5 adults) | 19/11/2015 |
| N_A_h3(43) | honeybees | no_varroa | apiary | H16_KCc47 | Kauai | Community college | *Apis mellifera* | 30mg (5 adults) | 8/10/2016 |
| N_A_h4(64) | honeybees | no_varroa | apiary | H16_KCc48 | Kauai | Community college | *Apis mellifera* | 30mg (5 adults) | 8/10/2016 |
| N_A_h5(128) | honeybees | no_varroa | apiary | H16_KCc49 | Kauai | Community college | *Apis mellifera* | 30mg (5 adults) | 8/10/2016 |
| N_A_h6(129) | honeybees | no_varroa | apiary | H16_KDp2 | Kauai | Koloa Dave Paniolo | *Apis mellifera* | 30mg (5 adults) | 8/10/2016 |
| N_W_h1(4) | honeybees | no_varroa | non-apiray | H15_Mu1a | Molokai | USDA Plant centre | *Apis mellifera* | 30mg (5 adults) | 19/11/2015 |
| N_W_h2(65) | honeybees | no_varroa | non-apiray | H16_KCl2 | Kauai | Canyon lookout | *Apis mellifera* | 30mg (5 adults) | 7/10/2016 |
| V_A_b1(5) | solitary_bees | w_varroa | apiary | H15_Bg37 | Big Island | Garnett | *Megachile chlorura* | 1 adult | 21/11/2015 |
| V_A_b2(6) | solitary_bees | w_varroa | apiary | H15_Op7 | Oahu | Pearl City - Urban Garden Centre | *Chalicodoma umbipennis* | 1 adult | 24/11/2015 |
| V_A_b3(66) | solitary_bees | w_varroa | apiary | H15_Op29 | Oahu | Pearl City - Urban Garden Centre | *Ceratina smaragdula* | 1 adult | 24/11/2015 |
| V_A_b4(130) | solitary_bees | w_varroa | apiary | H15_Ou4 | Oahu | UH | *Ceratina smaragdula* | 1 adult | 18/11/2015 |
| V_W_b1(7) | solitary_bees | w_varroa | non-apiray | H15_Ba6 | Big Island | Amy Greenwell Botanical Garden | *Megachile chlorura* | 1 adult | 21/11/2015 |
| V_W_b2(8) | solitary_bees | w_varroa | non-apiray | H15_Og7 | Oahu | Waikiki - Urban garden plots | *Chalicodoma umbipennis* | 1 adult | 25/11/2015 |
| V_W_b3(67) | solitary_bees | w_varroa | non-apiray | H15_Oc15 | Oahu | Coco head | *Chalicodoma umbipennis* | 1 adult | 17/11/2015 |
| V_W_b4(131) | solitary_bees | w_varroa | non-apiray | H16_OCc10 | Oahu | Coco head | *Hylaeus strenuus* | 1 adult | 10/10/2016 |
| N_A_b1(44) | solitary_bees | no_varroa | apiary | H16_KCc4 | Kauai | Community college | *Chalicodoma umbipennis* | 1 adult | 8/10/2016 |
| N_A_b2(68) | solitary_bees | no_varroa | apiary | H16_KCc5 | Kauai | Community college | *Chalicodoma umbipennis* | 1 adult | 8/10/2016 |
| N_A_b3(69) | solitary_bees | no_varroa | apiary | H16_KCc6 | Kauai | Community college | *Chalicodoma umbipennis* | 1 adult | 8/10/2016 |
| N_A_b4(103) | solitary_bees | no_varroa | apiary | H16_KCc7 | Kauai | Community college | *Megachile chlorura* | 1 adult | 8/10/2016 |
| N_W_b1(15) | solitary_bees | no_varroa | non-apiray | H15_Mu18 | Molokai | USDA Plant centre | *Chalicodoma umbipennis* | 1 adult | 19/11/2015 |
| N_W_b2(10) | solitary_bees | no_varroa | non-apiray | H15_Mu19 | Molokai | USDA Plant centre | *Megachile chlorura* | 0.5 adult | 19/11/2015 |
| N_W_b3(70) | solitary_bees | no_varroa | non-apiray | H15_Mu37 | Molokai | USDA Plant centre | *Ceratina smaragdula* | 1 adult | 19/11/2015 |
| N_W_b4(104) | solitary_bees | no_varroa | non-apiray | H16_Kp2 | Kauai | Kilauea pt - lighthouse | *Hylaeus connectens* | 1 adult | 8/10/2016 |
| V_A_v1(18) | V_pensylvanica | w_varroa | apiary | H15_Bh1 | Big Island | Honokaa | *Vespula pensylvanica* | 0.5 adult | 22/11/2015 |
| V_A_v2(40) | V_pensylvanica | w_varroa | apiary | H15_Bh2 | Big Island | Honokaa | *Vespula pensylvanica* | 0.5 adult | 22/11/2015 |
| V_A_v3(57) | V_pensylvanica | w_varroa | apiary | H15_Bh3 | Big Island | Honokaa | *Vespula pensylvanica* | 0.5 adult | 22/11/2015 |
| V_A_v4(98) | V_pensylvanica | w_varroa | apiary | H15_Bh4 | Big Island | Honokaa | *Vespula pensylvanica* | 0.5 adult | 22/11/2015 |
| V_W_v1(19) | V_pensylvanica | w_varroa | non-apiray | H15_Bke1 | Big Island | Keolamauloa | *Vespula pensylvanica* | 0.5 adult | 22/11/2015 |
| V_W_v2(41) | V_pensylvanica | w_varroa | non-apiray | H15_Bke2 | Big Island | Keolamauloa | *Vespula pensylvanica* | 0.5 adult | 22/11/2015 |
| V_W_v3(58) | V_pensylvanica | w_varroa | non-apiray | H15_Bke3 | Big Island | Keolamauloa | *Vespula pensylvanica* | 0.5 adult | 21/11/2015 |
| V_W_v4(99) | V_pensylvanica | w_varroa | non-apiray | H15_Bke4 | Big Island | Keolamauloa | *Vespula pensylvanica* | 0.5 adult | 21/11/2015 |
| N_W_v1(59) | V_pensylvanica | no_varroa | non-apiray | H16_Kw2 | Kauai | Waimea Canyon road | *Vespula pensylvanica* | 0.5 adult | 7/10/2016 |
| N_W_v2(60) | V_pensylvanica | no_varroa | non-apiray | H16_Kw3 | Kauai | Waimea Canyon road | *Vespula pensylvanica* | 0.5 adult | 7/10/2016 |
| N_W_v3(61) | V_pensylvanica | no_varroa | non-apiray | H16_Kw4 | Kauai | Waimea Canyon road | *Vespula pensylvanica* | 0.5 adult | 7/10/2016 |
| N_W_v4(100) | V_pensylvanica | no_varroa | non-apiray | H16_KWa2 | Kauai | Waimea Canyon road | *Vespula pensylvanica* | 0.5 adult | 7/10/2016 |
| N_W_v5(101) | V_pensylvanica | no_varroa | non-apiray | H16_KWa3 | Kauai | Waimea Canyon road | *Vespula pensylvanica* | 0.5 adult | 7/10/2016 |
| N_W_v6(120) | V_pensylvanica | no_varroa | non-apiray | H16_Kk3 | Kauai | Kokee - on trail | *Vespula pensylvanica* | 0.5 adult | 7/10/2016 |
| N_W_v7(121) | V_pensylvanica | no_varroa | non-apiray | H16_KCl1 | Kauai | Canyon lookout 2 | *Vespula pensylvanica* | 0.5 adult | 7/10/2016 |
| N_W_v8(122) | V_pensylvanica | no_varroa | non-apiray | H16_KCt1 | Kauai | Canyon trai lhead | *Vespula pensylvanica* | 0.5 adult | 7/10/2016 |
| V_A_d1(34) | potter_wasps | w_varroa | apiary | H15_Bg20 | Big Island | Garnett | *Delta campaniforme ensuriens* | 0.5 adult | 21/11/2015 |
| V_A_d2(20) | potter_wasps | w_varroa | apiary | H15_Bg21 | Big Island | Garnett | *Delta campaniforme ensuriens* | 0.5 adult | 21/11/2015 |
| V_A_d3(93) | potter_wasps | w_varroa | apiary | H15_Op17 | Oahu | Pearl City - Urban Garden Centre | *Delta pyriforme philippinese* | 0.25 adult | 24/11/2015 |
| V_A_d4(113) | potter_wasps | w_varroa | apiary | H15_Opa2 | Oahu | Howard - Party | *Delta campaniforme esuriens* | 0.5 adult | 24/11/2015 |
| V_W_d1(35) | potter_wasps | w_varroa | non-apiray | H15_Og3 | Oahu | Waikiki - Urban garden plots | *Delta campaniforme esuriens* | 0.5 adult | 25/11/2015 |
| V_W_d2(16) | potter_wasps | w_varroa | non-apiray | H15_Og4 | Oahu | Waikiki - Urban garden plots | *Delta campaniforme esuriens* | 0.5 adult | 25/11/2015 |
| V_W_d3(94) | potter_wasps | w_varroa | non-apiray | H16_OCc21 | Oahu | Coco head | *Delta pyriforme philippinense* | 0.25 adult | 10/10/2016 |
| V_W_d4(114) | potter_wasps | w_varroa | non-apiray | H16_OCc17 | Oahu | Coco head | *Delta pyriforme philippinense* | 0.25 adult | 10/10/2016 |
| N_A_d1(51) | potter_wasps | no_varroa | apiary | H16_KCc39 | Kauai | Community college | *Delta pyriforme philippinense* | 0.25 adult | 6/10/2016 |
| N_A_d2(95) | potter_wasps | no_varroa | apiary | H16_KCc40 | Kauai | Community college | *Delta pyriforme philippinense* | 0.25 adult | 6/10/2016 |
| N_A_d3(96) | potter_wasps | no_varroa | apiary | H16_KCc41 | Kauai | Community college | *Delta campaniforme ensuriens* | 0.5 adult | 6/10/2016 |
| N_A_d3(115) | potter_wasps | no_varroa | apiary | H16_KCc42 | Kauai | Community college | *Delta campaniforme ensuriens* | 0.5 adult | 6/10/2016 |
| N_W_d1(36) | potter_wasps | no_varroa | non-apiray | H15_Mm1 | Molokai | Molokai burger | *Delta campaniform ensuriens* | 0.5 adult | 19/11/2015 |
| N_W_d2(52) | potter_wasps | no_varroa | non-apiray | H15_Mf10 | Molokai | Farm - CTAHR | *Delta campaniform ensuriens* | 0.5 adult | 19/11/2015 |
| N_W_d3(97) | potter_wasps | no_varroa | non-apiray | H15_Mf11 | Molokai | Farm - CTAHR | *Delta campaniform ensuriens* | 0.5 adult | 19/11/2015 |
| N_W_d4(116) | potter_wasps | no_varroa | non-apiray | H16_Kp1 | Kauai | Kilauea pt lighthouse | *Chalybion bengalense* | 1 adult | 8/10/2016 |
| V_A_m1(30) | sol_wasps | w_varroa | apiary | H15_Bg30 | Big Island | Garnett | *Pachodynerus nasidens* | 0.5 adult | 21/11/2015 |
| V_A_m2(47) | sol_wasps | w_varroa | apiary | H15_Bg33 | Big Island | Garnett | *Ichneumonidae* | 1 adult | 21/11/2015 |
| V_A_m3(74) | sol_wasps | w_varroa | apiary | H15_Ow6 | Oahu | Waimanalo | *Sceliphron caementarium* | 0.5 adult | 24/11/2015 |
| V_A_m4(109) | sol_wasps | w_varroa | apiary | H15_Ou12 | Oahu | UH | *Pachodynerus nasidens* | 1 adult | 18/11/2015 |
| V_W_m1(31) | sol_wasps | w_varroa | non-apiray | H15_Og13 | Oahu | Waikiki - Urban garden plots | *Pachodynerus nasidens* | 1 adult | 25/11/2015 |
| V_W_m2(48) | sol_wasps | w_varroa | non-apiray | H16_OCc3 | Oahu | Coco head | *Ampulex compressa* | 0.5 adult | 10/10/2016 |
| V_W_m3(75) | sol_wasps | w_varroa | non-apiray | H16_OCc5 | Oahu | Coco head | *Ampulex compressa* | 0.5 adult | 10/10/2016 |
| V_W_m4(110) | sol_wasps | w_varroa | non-apiray | H16_OCc7 | Oahu | Coco head | *Evania appendigaster* | 1 adult | 10/10/2016 |
| N_A_m1(32) | sol_wasps | no_varroa | apiary | H15_Mb10 | Molokai | Brenda | *Misc* | 1 adult | 19/11/2015 |
| N_A_m2(49) | sol_wasps | no_varroa | apiary | H16_KCc2 | Kauai | Community college | *Liris aurulenta* | 1 adult | 8/10/2016 |
| N_A_m3(76) | sol_wasps | no_varroa | apiary | H16_KCc37 | Kauai | Community college | *Ampulex compressa* | 0.5 adult | 6/10/2016 |
| N_A_m4(111) | sol_wasps | no_varroa | apiary | H16_KCc30 | Kauai | Community college | *Sceliphron caementarium* | 0.5 adult | 6/10/2016 |
| N_W_m1(33) | sol_wasps | no_varroa | non-apiray | H15_Mf14 | Molokai | Farm - CTAHR | *Chalybion bengalense* | 1 adult | 19/11/2015 |
| N_W_m2(50) | sol_wasps | no_varroa | non-apiray | H15_Mf15 | Molokai | Farm - CTAHR | *Chalybion bengalense* | 1 adult | 19/11/2015 |
| N_W_m3(77) | sol_wasps | no_varroa | non-apiray | H16_KWa1 | Kauai | Waimea Canyon Road | *Pachodynerus nasidens* | 1 adult | 7/10/2016 |
| N_W_m4(112) | sol_wasps | no_varroa | non-apiray | H15_Mf21 | Molokai | Farm - CTAHR | *Pachodynerus nasidens* | 1 adult | 19/11/2015 |
| V_A_p1(37) | polistes_wasps | w_varroa | apiary | H15_Bg10 | Big Island | Garnett | *polistes aurifer* | 0.5 adult | 21/11/2015 |
| V_A_p2(53) | polistes_wasps | w_varroa | apiary | H15_Bg11 | Big Island | Garnett | *polistes aurifer* | 0.5 adult | 21/11/2015 |
| V_A_p3(78) | polistes_wasps | w_varroa | apiary | H15_Op28 | Oahu | Pearl City - Urban Garden Centre | Polistes exclamens | 0.5 adult | 24/11/2015 |
| V_A_p4(117) | polistes_wasps | w_varroa | apiary | H16_OW3 | Oahu | Waimanalo | *Polistes aurifer* | 0.5 adult | 31/03/2016 |
| V_W_p1(38) | polistes_wasps | w_varroa | non-apiray | H15_Bw1 | Big Island | Waipio lookout | *Polistes aurifer* | 0.5 adult | 22/11/2015 |
| V_W_p2(54) | polistes_wasps | w_varroa | non-apiray | H16_OCc11 | Oahu | Coco head | *Polistes exclemans* | 0.5 adult | 10/10/2016 |
| V_W_p3(79) | polistes_wasps | w_varroa | non-apiray | H16_OCc12 | Oahu | Coco head | *Polistes exclemans* | 0.5 adult | 10/10/2016 |
| V_W_p4(118) | polistes_wasps | w_varroa | non-apiray | H16_OCc13 | Oahu | Coco head | *Polistes exclemans* | 0.5 adult | 10/10/2016 |
| N_A_p1(39) | polistes_wasps | no_varroa | apiary | H15_Mb8 | Molokai | Brenda | *Polistes aurides* | 0.5 adult | 19/11/2015 |
| N_A_p2(55) | polistes_wasps | no_varroa | apiary | H16_KCc32 | Kauai | Community college | *Polistes olivaceus* | 0.5 adult | 6/10/2016 |
| N_A_p3(80) | polistes_wasps | no_varroa | apiary | H16_KCc33 | Kauai | Community college | *Polistes olivaceus* | 0.5 adult | 6/10/2016 |
| N_A_p4(119) | polistes_wasps | no_varroa | apiary | H16_KCc34 | Kauai | Community college | *Polistes olivaceus* | 0.5 adult | 6/10/2016 |
| N_W_p1(17) | polistes_wasps | no_varroa | non-apiray | H15_Mu8 | Molokai | USDA Plant centre | *Polistes olivaceus* | 0.5 adult | 19/11/2015 |
| N_W_p2(56) | polistes_wasps | no_varroa | non-apiray | H15_Mf8 | Molokai | Farm - CTAHR | *Polistes esclamans* | 0.5 adult | 19/11/2015 |
| N_W_p3(81) | polistes_wasps | no_varroa | non-apiray | H15_Mf9 | Molokai | Farm - CTAHR | *Polistes esclamans* | 0.5 adult | 19/11/2015 |
| N_W_p4(102) | polistes_wasps | no_varroa | non-apiray | H16_KA1 | Kauai | Ag land kekaha | *Polistes aurifer* | 0.5 adult | 7/10/2016 |
| V_A_a1(22) | ants | w_varroa | apiary | H15_Op16 | Oahu | Pearl City - Urban Garden Centre | *Anoplolepis gracilipes* | 1 adult queen | 24/11/2015 |
| V_A_a2(23) | ants | w_varroa | apiary | H15_Op24 | Oahu | Pearl City - Urban Garden Centre | *Anoplolepis gracilipes* | 5 adults | 24/11/2015 |
| V_A_a3(89) | ants | w_varroa | apiary | H15_Ou3 | Oahu | UH | *Pheidole megacephala* | 5 adults | 16/11/2015 |
| V_A_a4(105) | ants | w_varroa | apiary | H16_Opat1 | Oahu | Pats-UH | *Pseudomyrmex gracilis* | 1 adult | 17/10/2016 |
| V_W_a1(24) | ants | w_varroa | non-apiray | H15_Oc2 | Oahu | Coco head | *Pseudomyrmex gracilis* | 1 adult | 17/11/2015 |
| V_W_a2(25) | ants | w_varroa | non-apiray | H16_OWR2 | Oahu | Waahila Ridge | *Anoplolepis gracilipes* | 5 adults | 7/04/2016 |
| V_W_a3(90) | ants | w_varroa | non-apiray | H16_OWR3 | Oahu | Waahila Ridge | *Anoplolepis gracilipes* | 5 adults | 7/04/2016 |
| V_W_a4(106) | ants | w_varroa | non-apiray | H16_OCc4 | Oahu | Coco head | *Anoplolepis gracilipes* | 1 adult | 10/10/2016 |
| N_A_a1(26) | ants | no_varroa | apiary | H15_Mb4 | Molokai | Brenda | *Camponotus variegatus* | 1 adult | 19/11/2015 |
| N_A_a2(27) | ants | no_varroa | apiary | H15_Mb6 | Molokai | Brenda | *Tapinomea melanocephalum* | 5 adults | 19/11/2015 |
| N_A_a3(91) | ants | no_varroa | apiary | H15_Mb9 | Molokai | Brenda | *Camponotus variegatus* | 1 adult | 19/11/2015 |
| N_A_a4(107) | ants | no_varroa | apiary | H16_KDp1 | Kauai | Koloa Dave Paniolo | *Pheidole megacephala* | 5 adults | 8/10/2016 |
| N_W_a1(28) | ants | no_varroa | non-apiray | H15_Mu3 | Molokai | USDA Plant centre | *Pheidole megacephala* | 5 adults | 19/11/2015 |
| N_W_a2(29) | ants | no_varroa | non-apiray | H15_Ma1 | Molokai | airport | *Pheidole megacephala* | 5 adults | 19/11/2015 |
| N_W_a3(92) | ants | no_varroa | non-apiray | H15_Mf1 | Molokai | Farm - CTAHR | *Technomyrmex albipennis* | 5 adults | 19/11/2015 |
| N_W_a4(108) | ants | no_varroa | non-apiray | H15_Mu4 | Molokai | USDA Plant centre | *Pheidole megacephala* | 5 adults | 19/11/2015 |
| V_A_f1(11) | flies | w_varroa | apiary | H15_Bg34 | Big Island | Garnett | *Syrphid sp* | 1 adult | 21/11/2015 |
| V_A_f2(45) | flies | w_varroa | apiary | H15_Ow13 | Oahu | Waimanalo | *Syrphidae* | 1 adult | 24/11/2015 |
| V_A_f3(71) | flies | w_varroa | apiary | H15_Ow14 | Oahu | Waimanalo | *Allograpta sp* | 1 adult | 24/11/2015 |
| V_A_f4(83) | flies | w_varroa | apiary | H15_Ou24 | Oahu | UH | *Allograpta sp* | 1 adult | 18/11/2015 |
| V_W_f1(12) | flies | w_varroa | non-apiray | H15_Ba7 | Big Island | Amy Greenwell Botanical Garden | *Syrphid sp* | 1 adult | 21/11/2015 |
| V_W_f2(13) | flies | w_varroa | non-apiray | H15_Ba10 | Big Island | Amy Greenwell Botanical Garden | *Allograpta sp* | 1 adult | 21/11/2015 |
| V_W_f3(72) | flies | w_varroa | non-apiray | H15_Oc25 | Oahu | Coco head | *Allograpta sp* | 1 adult | 17/11/2015 |
| V_W_f4(84) | flies | w_varroa | non-apiray | H15_Oc24 | Oahu | Coco head | *Allograpta sp* | 1 adult | 17/11/2015 |
| N_A_f1(46) | flies | no_varroa | apiary | H16_KCc3 | Kauai | Community college | *symopsyrphus sp* | 1 adult | 8/10/2016 |
| N_A_f2(73) | flies | no_varroa | apiary | H16_KCc43 | Kauai | Community college | *symopsyrphus sp* | 1 adult | 8/10/2016 |
| N_A_f3(85) | flies | no_varroa | apiary | H16_KCc44 | Kauai | Community college | *symopsyrphus sp* | 1 adult | 8/10/2016 |
| N_A_f4(86) | flies | no_varroa | apiary | H16_KCc45 | Kauai | Community college | *symopsyrphus sp* | 1 adult | 8/10/2016 |
| N_W_f1(14) | flies | no_varroa | non-apiray | H15_Ml1 | Molokai | lookout spot | *Allograpta sp* | 1 adult | 19/11/2015 |
| N_W_f2(9) | flies | no_varroa | non-apiray | H15_Mu21 | Molokai | USDA Plant centre | *Syrphid sp* | 1 adult | 19/11/2015 |
| N_W_f3(87) | flies | no_varroa | non-apiray | H16_Ku4 | Kauai | UH ag station Kapaa | *symosyrphus sp.* | 1 adult | 6/10/2015 |
| N_W_f4(88) | flies | no_varroa | non-apiray | H16_Kw5 | Kauai | Waimea Canyon Road | *Allograpta sp* | 1 adult | 7/10/2015 |
