# Supplementary material for: RNAseq of Deformed Wing Virus and Other Honey Bee-Associated Viruses in Eight Insect Taxa with or without Varroa Infestation
Source: Viruses. 2020 Oct 29;12(11):1229. doi: 10.3390/v12111229 (PMC7692275; doi:10.3390/v12111229)
Supplement: Supplementary file 1 [file viruses-12-01229-s001.zip › Supplementary_v2/Supp_table_s2_v2.docx]

**Supplementary Table S2**. RNAseq library information for individual samples presented according to pooling strategy.

| **Sample ID** | **Index #** | **Lane** | **Total raw reads (R1+R2)** | **Total clean reads (R1+R2+R0)** | | **Total clean reads (R1)** | **Total virus-derived reads (R1)** |
| --- | --- | --- | --- | --- | --- | --- | --- |
| N_A_h1 | 3 | 1 | 6779994 | 6882422 | 3339657 | | 159095 |
| N_W_b1 | 15 | 1 | 10714449 | 10889210 | 5271565 | | 51795 |
| N_W_b2 | 10 | 1 | 35612560 | 35945042 | 17642878 | | 149007 |
| N_W_f1 | 14 | 1 | 60428173 | 61075110 | 29893358 | | 559432 |
| N_W_f2 | 9 | 1 | 45044591 | 45783174 | 22156240 | | 409811 |
| N_W_h1 | 4 | 1 | 22289164 | 22581572 | 11000392 | | 498357 |
| N_W_p1 | 18 | 1 | 44453059 | 44891556 | 22009517 | | 383338 |
| V_A_b1 | 5 | 1 | 20156092 | 20495634 | 9910955 | | 489447 |
| V_A_b2 | 6 | 1 | 4078780 | 4140758 | 2008704 | | 71639 |
| V_A_d2 | 21 | 1 | 68489546 | 69337256 | 33823756 | | 344814 |
| V_A_f1 | 11 | 1 | 43956064 | 44601558 | 21658202 | | 310951 |
| V_A_h1 | 1 | 1 | 48131703 | 48832084 | 23719323 | | 21295818 |
| V_A_v1 | 19 | 1 | 24475473 | 24828814 | 12062567 | | 257569 |
| V_W_b1 | 7 | 1 | 17852095 | 18026096 | 8839760 | | 172278 |
| V_W_b2 | 8 | 1 | 3553142 | 3615536 | 1745798 | | 76745 |
| V_W_d2 | 16 | 1 | 45239372 | 45660016 | 22411575 | | 243340 |
| V_W_f1 | 12 | 1 | 61328286 | 62107034 | 30278092 | | 414113 |
| V_W_f2 | 13 | 1 | 46453296 | 47061372 | 22926101 | | 307886 |
| V_W_h1 | 2 | 1 | 10239734 | 10367980 | 5057364 | | 2618619 |
| V_W_v1 | 20 | 1 | 50470040 | 51027994 | 24958910 | | 585619 |
| Nextlib_L001(29) | N702 | 1 | * | * | 5086082 | | 617 |
| N_A_a1 | 5 | 2 | 17754647 | 17900944 | 8813150 | | 203523 |
| N_A_a2 | 6 | 2 | 46803051 | 47150598 | 23237901 | | 751383 |
| N_A_m1 | 10 | 2 | 31414390 | 31700490 | 15589157 | | 309142 |
| N_A_p1 | 18 | 2 | 30532844 | 30788330 | 15146627 | | 621852 |
| N_W_a1 | 7 | 2 | 45080220 | 45460410 | 22361783 | | 942862 |
| N_W_a2 | 22 | 2 | 45123415 | 45504402 | 22372928 | | 2188958 |
| N_W_d1 | 14 | 2 | 25837036 | 26025964 | 12826306 | | 370376 |
| N_W_m1 | 11 | 2 | 34556712 | 34901252 | 17111057 | | 232337 |
| V_A_a1 | 1 | 2 | 22081119 | 22315638 | 10939999 | | 428863 |
| V_A_a2 | 2 | 2 | 35254222 | 35553002 | 17491050 | | 600797 |
| V_A_d1 | 12 | 2 | 24973936 | 25172874 | 12391857 | | 464002 |
| V_A_m1 | 8 | 2 | 23598502 | 23822928 | 11689972 | | 734856 |
| V_A_p1 | 15 | 2 | 9376185 | 9448162 | 4655255 | | 213276 |
| V_A_v2 | 21 | 2 | 26176992 | 26382390 | 12986943 | | 629903 |
| V_W_a1 | 3 | 2 | 29137717 | 29409396 | 14438289 | | 671081 |
| V_W_a2 | 4 | 2 | 34233562 | 34540836 | 16971911 | | 769336 |
| V_W_d1 | 13 | 2 | 30757212 | 31021956 | 15252304 | | 616848 |
| V_W_m1 | 9 | 2 | 29316527 | 29629130 | 14508389 | | 522844 |
| V_W_p1 | 16 | 2 | 40501338 | 40859818 | 20076528 | | 1003387 |
| V_W_v2 | 20 | 2 | 34934875 | 35164584 | 17354035 | | 857904 |
| N_A_b1 | 12 | 3 | 16543195 | 16746406 | 8171732 | | 278197 |
| N_A_d1 | 1 | 3 | 27298278 | 27655940 | 13471767 | | 386620 |
| N_A_f1 | 28 | 3 | 29440896 | 29763100 | 14560828 | | 391022 |
| N_A_h2 | 17 | 3 | 26002377 | 26238248 | 12885974 | | 253216 |
| N_A_h3 | 24 | 3 | 18557045 | 18832444 | 9142048 | | 584380 |
| N_A_m2 | 32 | 3 | 29166128 | 29529130 | 14404346 | | 107120 |
| N_A_p2 | 5 | 3 | 20447386 | 20768372 | 10065798 | | 513898 |
| N_W_d2 | 2 | 3 | 41526862 | 42039678 | 20508517 | | 377127 |
| N_W_m2 | 33 | 3 | 30767969 | 31183638 | 15177751 | | 70331 |
| N_W_p2 | 6 | 3 | 28339835 | 28715618 | 13984668 | | 523226 |
| N_W_v1 | 8 | 3 | 24466583 | 24877004 | 12035780 | | 830567 |
| N_W_v2 | 9 | 3 | 27202173 | 27621716 | 13394605 | | 825738 |
| N_W_v3 | 10 | 3 | 26412900 | 26804520 | 13014335 | | 1006164 |
| V_A_f2 | 26 | 3 | 29642224 | 29982236 | 14651999 | | 360236 |
| V_A_m2 | 29 | 3 | 32972656 | 33321024 | 16313231 | | 196763 |
| V_A_p2 | 3 | 3 | 30400712 | 30836768 | 14987624 | | 334067 |
| V_A_v3 | 11 | 3 | 37426266 | 38123568 | 18365928 | | 7690413 |
| V_W_m2 | 31 | 3 | 36918074 | 37403788 | 18219176 | | 1986000 |
| V_W_p2 | 4 | 3 | 23485010 | 23749254 | 11613369 | | 770419 |
| V_W_v3 | 7 | 3 | 30892251 | 31301350 | 15243775 | | 573709 |
| Nextlib_L003(29) | N702 | 3 | * | * | 4821234 | | 714 |
| control_ex_1(82) | 28 | 4 | 3379968 | 3403758 | 1679285 | | 65021 |
| N_A_b2 | 12 | 4 | 20872737 | 20970882 | 10387949 | | 241415 |
| N_A_b3 | 20 | 4 | 5413155 | 5450398 | 2688223 | | 279364 |
| N_A_f2 | 8 | 4 | 40850183 | 41090336 | 20306400 | | 449343 |
| N_A_h4 | 1 | 4 | 23534705 | 23765408 | 11654794 | | 654174 |
| N_A_m3 | 10 | 4 | 39357941 | 39552224 | 19585700 | | 287232 |
| N_A_p3 | 16 | 4 | 47370176 | 47660408 | 23543451 | | 836439 |
| N_W_b3 | 5 | 4 | 40156233 | 40389816 | 19966258 | | 196627 |
| N_W_h2 | 2 | 4 | 25706753 | 25889468 | 12764894 | | 816171 |
| N_W_m3 | 13 | 4 | 41785127 | 42036472 | 20768898 | | 818442 |
| N_W_p3 | 18 | 4 | 34452660 | 34625294 | 17141512 | | 415533 |
| V_A_b3 | 3 | 4 | 40837008 | 41101878 | 20291555 | | 266869 |
| V_A_f3 | 6 | 4 | 44373111 | 44621554 | 22064621 | | 351935 |
| V_A_h2 | 19 | 4 | 26491043 | 26666540 | 13158865 | | 173639 |
| V_A_m3 | 21 | 4 | 44896306 | 45209178 | 22292904 | | 163151 |
| V_A_p3 | 14 | 4 | 44995749 | 45243048 | 22376790 | | 773158 |
| V_W_b3 | 4 | 4 | 33932138 | 34145198 | 16863438 | | 416764 |
| V_W_f3 | 7 | 4 | 44434398 | 44681500 | 22096217 | | 446229 |
| V_W_h2 | 11 | 4 | 41098486 | 41441696 | 20379729 | | 2463755 |
| V_W_m3 | 9 | 4 | 11140035 | 11206244 | 5537583 | | 106494 |
| V_W_p3 | 15 | 4 | 35520062 | 35732258 | 17656901 | | 1038396 |
| Nextlib_L004(29) | N702 | 4 | * | * | 3884462 | | 560 |
| N_A_a3 | 45 | 5 | 19119171 | 19336612 | 9451092 | | 356546 |
| N_A_d2 | 17 | 5 | 24271998 | 24528496 | 12008339 | | 546537 |
| N_A_d3 | 24 | 5 | 34516509 | 34906430 | 17064060 | | 402780 |
| N_A_f3 | 38 | 5 | 40535659 | 40936184 | 20068290 | | 369683 |
| N_A_f4 | 39 | 5 | 29196606 | 29541442 | 14426646 | | 317926 |
| N_W_a3 | 46 | 5 | 24625763 | 24902630 | 12174973 | | 4360478 |
| N_W_d3 | 26 | 5 | 27990690 | 28277718 | 13852411 | | 220044 |
| N_W_f3 | 40 | 5 | 34445367 | 34828452 | 17033471 | | 271506 |
| N_W_f4 | 41 | 5 | 39815998 | 40404982 | 19614818 | | 748620 |
| N_W_p4 | 33 | 5 | 23360900 | 23661728 | 11530583 | | 539262 |
| N_W_v4 | 21 | 5 | 32805003 | 33499558 | 16056561 | | 1316282 |
| N_W_v5 | 22 | 5 | 15798953 | 16059036 | 7769942 | | 619583 |
| V_A_a3 | 43 | 5 | 33837848 | 34202402 | 16737677 | | 116684 |
| V_A_d3 | 47 | 5 | 38278559 | 38675810 | 18941453 | | 439255 |
| V_A_f4 | 30 | 5 | 31333315 | 31684502 | 15491615 | | 167323 |
| V_A_v4 | 28 | 5 | 24877950 | 25181540 | 12287940 | | 515995 |
| V_W_a3 | 44 | 5 | 37977280 | 38359180 | 18800096 | | 372857 |
| V_W_d3 | 48 | 5 | 27527740 | 27847684 | 13604632 | | 574881 |
| V_W_f4 | 37 | 5 | 39291162 | 39774990 | 19404359 | | 473077 |
| V_W_v4 | 20 | 5 | 35727354 | 36135546 | 17660353 | | 7246266 |
| control_ex_2(123) | 39 | 6 | 618234 | 628868 | 304134 | | 6777 |
| N_A_a4 | 5 | 6 | 5441013 | 5541276 | 2673155 | | 29168 |
| N_A_b4 | 1 | 6 | 3243837 | 3316548 | 1589666 | | 49625 |
| N_A_m4 | 8 | 6 | 8439112 | 8564650 | 4157472 | | 33019 |
| N_A_p4 | 33 | 6 | 4590911 | 4664088 | 2259165 | | 91626 |
| N_W_a4 | 36 | 6 | 4671598 | 4745206 | 2299320 | | 225308 |
| N_W_b4 | 3 | 6 | 5711762 | 5801958 | 2816249 | | 35407 |
| N_W_d4 | 42 | 6 | 7095282 | 7212068 | 3489600 | | 11777 |
| N_W_m4 | 9 | 6 | 6870550 | 7017116 | 3377758 | | 76866 |
| N_W_v6 | 23 | 6 | 7278711 | 7403096 | 3578146 | | 194744 |
| N_W_v7 | 34 | 6 | 6663335 | 6799644 | 3269615 | | 165542 |
| N_W_v8 | 35 | 6 | 5120561 | 5218208 | 2511985 | | 164197 |
| V_A_a4 | 3 | 6 | 5308798 | 5406844 | 2609147 | | 31083 |
| V_A_d4 | 10 | 6 | 7649645 | 7764326 | 3767944 | | 73631 |
| V_A_m4 | 6 | 6 | 7734665 | 7854094 | 3809164 | | 70433 |
| V_A_p4 | 29 | 6 | 8136647 | 8241328 | 4016553 | | 87322 |
| V_W_a4 | 4 | 6 | 7111871 | 7228506 | 3515226 | | 148031 |
| V_W_d4 | 11 | 6 | 7138353 | 7271834 | 3502830 | | 304003 |
| V_W_m4 | 7 | 6 | 6705790 | 6821640 | 3298475 | | 41328 |
| V_W_p4 | 31 | 6 | 4751029 | 4904104 | 2336046 | | 120504 |
| N_A_h5 | 5 | 7 | 16894318 | 17031652 | 8378925 | | 511509 |
| N_A_h6 | 6 | 7 | 28565730 | 28797486 | 14167416 | | 1241120 |
| V_A_b4 | 7 | 7 | 18136315 | 18266484 | 9003428 | | 122420 |
| V_A_h3 | 1 | 7 | 29570928 | 29881382 | 14634132 | | 5380218 |
| V_A_h4 | 2 | 7 | 31656696 | 31906618 | 15703813 | | 14006631 |
| V_W_b4 | 8 | 7 | 7727170 | 7885826 | 3784476 | | 314242 |
| V_W_h3 | 3 | 7 | 35448553 | 35774976 | 17565046 | | 9590800 |
| V_W_h4 | 4 | 7 | 37973529 | 38255404 | 18846347 | | 16699812 |
| Nextlib_L007(29) | N702 | 7 | * | * | 3942118 | | 426 |
| Nextlib_629L008(29) | N701 | 8# | 28163267 | 28744180 | 4637213 | | 551 |
| Nextlib_L008(29) | N702 | 8# | * | * | 6083241 | | 659 |

* Aliquots of the *E. coli* library “Nextlib_L00X” was used as a control across multiple lanes and summary statistics were only shown for all replicates combined (not shown)

# Lane 8 contained other honey bee samples not used in this study.
