# Supplementary material for: RNAseq of Deformed Wing Virus and Other Honey Bee-Associated Viruses in Eight Insect Taxa with or without Varroa Infestation
Source: Viruses. 2020 Oct 29;12(11):1229. doi: 10.3390/v12111229 (PMC7692275; doi:10.3390/v12111229)
Supplement: Supplementary file 1 [file viruses-12-01229-s001.zip › Supplementary_v2/Supp_table_s3_v3.docx]

**Supplementary table S3**. All honey bee-associated viruses tested for in this study.

|  | **Abbreviation** | **Accession number** | **reference** |
| --- | --- | --- | --- |
| Deformed wing virus type A | DWV-A | NC_004830.2 | [6] |
| Deformed wing virus type B/VDV-1 | DWV-B | AY251269 | [7] |
| Black queen cell virus | BQCV | NC_003784 | [57] |
| Sacbrood virus | SBV | NC_002066.1 | [58] |
| Israeli acute paralysis virus | IAPV | NC_009025.1 | [59] |
| Acute bee paralysis virus | ABPV | NC_002548.1 | [60] |
| Kashmir bee virus | KBV | NC_004807.1 | [60] |
| Chronic bee paralysis virus | CBPV | [NC_010711.1](https://www.ncbi.nlm.nih.gov/nuccore/NC_010711.1) | [61] |
|  |  | [NC_010712.1](https://www.ncbi.nlm.nih.gov/nuccore/NC_010712.1) |  |
| Slow bee paralysis virus | SBPV | NC_014137.1 | [62] |
| Lake Sinai virus-1 | LSV-1 | HQ871931 | [63] |
| Lake Sinai virus-2 | LSV-2 | HQ888865 | [63] |
| aphid lethal paralysis virus | ALPV |  |  |
|  |  | KJ817182.1 | [64] |
| Big Sioux River virus | BSRV | JF423195.1 | [63] |
|  |  | JF423196 |  |
|  |  | JF423197 |  |
|  |  | JF423198 |  |
| Tobacco ringspot virus | TRSV | [JQ710729](http://mbio.asm.org/external-ref?link_type=ncbi:nucleotide&access_num=JQ710729) | [65] |
|  |  | [JQ710730](http://mbio.asm.org/external-ref?link_type=ncbi:nucleotide&access_num=JQ710730) |  |
| Halictus scabiose Adikon virus | HsAV | Sequence obtained from within the reference. | [66] |
| Moku virus | MV | NC_031338.1 | [27] |
| Milolii virus | MiV | MF155030.1 | [28] |

**Additional supplementary references**

1. Leat, N.; Ball, B.; Govan, V.; Davison, S. Analysis of the complete genome sequence of black queen-cell virus, a picorna-like virus of honey bees. *J. Gen. Virol.* **2000**, *81*, 2111–2119, doi:10.1099/0022-1317-81-8-2111.
2. Ghosh, R.C.; Ball, B.V.; Willcocks, M.M.; Carter, M.J. The nucleotide sequence of sacbrood virus of the honey bee: An insect picorna-like virus. *J. Gen. Virol.* **1999**, *80*, 1541–1549, doi:10.1099/0022-1317-80-6-1541.
3. Maori, E.; Lavi, S.; Mozes-Koch, R.; Gantman, Y.; Peretz, Y.; Edelbaum, O.; Tanne, E.; Sela, I. Isolation and characterization of Israeli acute paralysis virus, a dicistrovirus affecting honeybees in Israel: evidence for diversity due to intra- and inter-species recombination. *J. Gen. Virol.* **2007**, *88*, 3428–3438, doi:10.1099/vir.0.83284-0.
4. De Miranda, J.R.; Drebot, M.; Tyler, S.; Shen, M.; Cameron, C.E.; Stoltz, D.B.; Camazine, S.M. Complete nucleotide sequence of Kashmir bee virus and comparison with acute bee paralysis virus. *J. Gen. Virol.* **2004**, *85*, 2263–2270, doi:10.1099/vir.0.79990-0.
5. Olivier, V.; Blanchard, P.; Chaouch, S.; Lallemand, P.; Schurr, F.; Celle, O.; Dubois, E.; Tordo, N.; Thiéry, R.; Houlgatte, R.; et al. Molecular characterisation and phylogenetic analysis of Chronic bee paralysis virus, a honey bee virus. *Virus Res.* **2008**, *132*, 59–68, doi:10.1016/j.virusres.2007.10.014.
6. De Miranda, J.R.; Dainat, B.; Locke, B.; Cordoni, G.; Berthoud, H.; Gauthier, L.; Neumann, P.; Budge, G.E.; Ball, B.V.; Stoltz, D.B. Genetic characterization of slow bee paralysis virus of the honeybee (*Apis mellifera* L.). *J. Gen. Virol.* **2010**, *91*, 2524–2530, doi:10.1099/vir.0.022434-0.
7. Runckel, C.; Flenniken, M.L.; Engel, J.C.; Ruby, J.G.; Ganem, D.; Andino, R.; DeRisi, J.L. Temporal Analysis of the Honey Bee Microbiome Reveals Four Novel Viruses and Seasonal Prevalence of Known Viruses, Nosema, and Crithidia. *PLoS ONE* **2011**, *6*, e20656, doi:10.1371/journal.pone.0020656.
8. Liu, S.; Vijayendran, D.; Carrillo-Tripp, J.; Miller, W.A.; Bonning, B.C. Analysis of new aphid lethal paralysis virus (ALPV) isolates suggests evolution of two ALPV species. *J. Gen. Virol.* **2014**, *95*, 2809–2819, doi:10.1099/vir.0.069765-0.
9. Li, J.L.; Cornman, R.S.; Evans, J.D.; Pettis, J.S.; Zhao, Y.; Murphy, C.; Peng, W.J.; Wu, J.; Hamilton, M.; Boncristiani, H.F.; et al. Systemic Spread and Propagation of a Plant-Pathogenic Virus in European Honeybees, *Apis mellifera*. *mBio* **2014**, *5*, e00898-13, doi:10.1128/mbio.00898-13.
10. Bigot, D.; Dalmon, A.; Roy, B.; Hou, C.; Germain, M.; Romary, M.; Deng, S.; Diao, Q.; Weinert, L.A.; Cook, J.M.; et al. The discovery of Halictivirus resolves the Sinaivirus phylogeny. *J. Gen. Virol.* **2017**, *98*, 2864–2875, doi:10.1099/jgv.0.000957.
