# Supplementary material for: RNAseq of Deformed Wing Virus and Other Honey Bee-Associated Viruses in Eight Insect Taxa with or without Varroa Infestation
Source: Viruses. 2020 Oct 29;12(11):1229. doi: 10.3390/v12111229 (PMC7692275; doi:10.3390/v12111229)
Supplement: Supplementary file 1 [file viruses-12-01229-s001.zip › Supplementary_v2/Supp_table_s4_v2.docx]

**Supplementary Table S4**. Summary for ANOVA testing for the individual and interactive effects of virus, *Varroa* status and taxon on viral abundance (RPKM) in insects in Hawaii.

| **Fixed effects** | **Df** | **P** | **Significance** |
| --- | --- | --- | --- |
| virus | 16 | < 2.2e-16 | *** |
| *Varroa*_status | 1 | 0.003386 | ** |
| taxon | 7 | 4.113e-12 | *** |
| virus:*Varroa*_status | 16 | 1.185e-15 | *** |
| virus:taxon | 112 | < 2.2e-16 | *** |
| *Varroa*_status:taxon | 7 | 2.555e-08 | *** |
| Virus: *Varroa* status: taxon | 112 | < 2.2e-16 | *** |
